# The Gcn2 Regulator Yih1 Interacts with the Cyclin Dependent Kinase Cdc28 and Promotes Cell Cycle Progression through G2/M in Budding Yeast

**DOI:** 10.1371/journal.pone.0131070

**Published:** 2015-07-15

**Authors:** Richard C. Silva, Martina Dautel, Bruno M. Di Genova, David C. Amberg, Beatriz A. Castilho, Evelyn Sattlegger

**Affiliations:** 1 Department of Microbiology, Immunology and Parasitology, Escola Paulista de Medicina, Universidade Federal de São Paulo, São Paulo, Brazil; 2 Institute of Natural and Mathematical Sciences, Massey University, Auckland, New Zealand; 3 Department of Biochemistry and Molecular Biology, Upstate Medical University, State University of New York, Syracuse, New York, United States of America; Fondazione Edmund Mach, Research and Innovation Centre, ITALY

## Abstract

The *Saccharomyces cerevisiae* protein Yih1, when overexpressed, inhibits the eIF2 alpha kinase Gcn2 by competing for Gcn1 binding. However, deletion of *YIH1* has no detectable effect on Gcn2 activity, suggesting that Yih1 is not a general inhibitor of Gcn2, and has no phenotypic defect identified so far. Thus, its physiological role is largely unknown. Here, we show that Yih1 is involved in the cell cycle. Yeast lacking Yih1 displays morphological patterns and DNA content indicative of a delay in the G2/M phases of the cell cycle, and this phenotype is independent of Gcn1 and Gcn2. Accordingly, the levels of phosphorylated eIF2α, which show a cell cycle-dependent fluctuation, are not altered in cells devoid of Yih1. We present several lines of evidence indicating that Yih1 is in a complex with Cdc28. Yih1 pulls down endogenous Cdc28 *in vivo* and this interaction is enhanced when Cdc28 is active, suggesting that Yih1 modulates the function of Cdc28 in specific stages of the cell cycle. We also demonstrate, by Bimolecular Fluorescence Complementation, that endogenous Yih1 and Cdc28 interact with each other, confirming Yih1 as a *bona fide* Cdc28 binding partner. Amino acid substitutions within helix H2 of the RWD domain of Yih1 enhance Yih1-Cdc28 association. Overexpression of this mutant, but not of wild type Yih1, leads to a phenotype similar to that of *YIH1* deletion, supporting the view that Yih1 is involved through Cdc28 in the regulation of the cell cycle. We further show that IMPACT, the mammalian homologue of Yih1, interacts with CDK1, the mammalian counterpart of Cdc28, indicating that the involvement with the cell cycle is conserved. Together, these data provide insights into the cellular function of Yih1/IMPACT, and provide the basis for future studies on the role of this protein in the cell cycle.

## Introduction

The gene encoding for the protein IMPACT was first identified in a screen for imprinted genes in mice [1]. IMPACT consists of a C-terminal domain that is conserved in all kingdoms of life, and hence its name (imprinted and ancient). IMPACT is also found in *S*. *cerevisiae* where it is called Yih1, for yeast IMPACT homologue [2].

The ancient domain of Yih1 was successfully modeled based on the structure of the ancient domain of the yigZ protein of *E*. *coli* [3]. Invariant sequence features present in both Yih1 and yigZ are located in loop regions clustered on the same side of the molecule, suggesting that these motifs may be involved in binding to molecules that are evolutionary conserved. However, despite the high conservation of this domain, its function and potential binding partners remain elusive.

The N-terminal portion of IMPACT/Yih1 harbors an RWD domain (present in RING finger proteins, WD-repeat containing proteins, and DEAD-like helicases) that shares similarities with the RWD domain found at the N-terminus of the protein kinase Gcn2 [2–4].

In all eukaryotes, Gcn2 senses amino acid starvation by binding uncharged tRNAs that accumulate when cells are deprived of amino acids, and then phosphorylates the alpha subunit of eukaryotic translation initiation factor 2 (eIF2α) [5]. This leads to reduced global protein synthesis and simultaneously to increased translation of transcriptional activators, Gcn4 in yeast and ATF4 in mammals, which increase expression of stress-remedial genes including those coding for amino acid biosynthetic enzymes and amino acid transporters, enabling cells to overcome amino acid starvation and to maintain cellular homeostasis. In addition to regulating protein synthesis, Gcn2 is also involved in the G1/S transition delay observed upon DNA damage in budding and fission yeast, and in G1 delay upon nutrient starvation [6–8]. In mammals, the Gcn2 signaling pathway has evolved to e.g. control memory formation, feeding behavior and plays a role in cancer progression [9–11].

For sensing uncharged tRNAs, the RWD domain of Gcn2 must directly bind to its effector protein Gcn1, an interaction that is essential for Gcn2 activation [12]. Based on this, it was proposed that IMPACT/Yih1 impairs the activation of Gcn2 by competing with the latter for binding to Gcn1 [2]. In fact, we have previously shown that both IMPACT and Yih1 bind to Gcn1 when overexpressed in yeast, and inhibit Gcn2 activity in response to diverse environmental stress stimuli [13–15]. In mammals, IMPACT is a cytoplasmic protein preferentially expressed in the brain, especially abundant in hypothalamic neurons [14, 16]. Similar to yeast, overexpression of IMPACT in mouse embryonic fibroblasts (MEFs) inhibits Gcn2 activation and eIF2α phosphorylation elicited by amino acid or glucose starvation, UV irradiation and proteasome inhibition [14, 15]. Conversely, knock down of IMPACT in neuron-like N2a cells, which express higher levels of IMPACT in comparison to non-neuronal cell lineages, results in stronger Gcn2 activation upon amino acid starvation. The basal activity of Gcn2 in differentiated N2a cells increases when IMPACT, whose expression is even higher in relation to their undifferentiated counterpart, is depleted. Interestingly, IMPACT promotes neurite outgrowth in N2a cells and this phenotype appears to be only partially dependent on Gcn2, suggesting that IMPACT may also be involved in Gcn2-independent pathways [17].

In yeast, however, deletion of *YIH1* does not have any detectable effect on Gcn2 activity in otherwise wild type cells, suggesting that Yih1 is not a general Gcn2 inhibitor. Interestingly, native Yih1 forms a heterodimeric complex with monomeric G-actin. Actin haploinsufficient yeast cells (*ACT1/act1Δ)* are impaired in overcoming amino acid starvation, and this can be partially reverted by deleting *YIH1*, suggesting that Yih1 released from G-actin inhibits Gcn2 under amino acid starvation [13]. It is possible that Yih1 regulates Gcn2 in specific locations in the cell and/or under certain conditions where maximal protein synthesis is required [13]. Despite intensive efforts to uncover a phenotypic defect of the lack of Yih1, none has been identified so far [13, 18]. Therefore, the conditions leading to Yih1-mediated Gcn2 inhibition and the physiological function of native Yih1 are still largely unknown.

Here, we describe that Yih1 is involved in the cell cycle. Yeast cells lacking Yih1 accumulate in the G2/M phases of the cell cycle. This phenotype is independent of Gcn2 and Gcn1, raising the possibility that Yih1 has a second role in the cells independent of Gcn2. Using several assays, we show that Yih1 physically interacts with Cdc28, a cyclin-dependent kinase essential for the progression of cells through the cell cycle. Yih1-Cdc28 interaction increases in the cell cycle phases where Cdc28 is known to be active, suggesting a potential functional role for this interaction in the regulation of cell cycle progression. We found that IMPACT binds to Cdc28 in yeast and to CDK1 in mammals, further strengthening the notion that Yih1 and IMPACT are functional homologues. Our findings provide a novel function for Yih1, as a modulator of the cell cycle.

## Materials and Methods

### Strains and plasmids

Yeast strains were grown as previously described [3, 13]. All yeast strains and plasmids used in this study are listed in S1 and S2 Tables, respectively. Strains for BiFC analyses were constructed following standard procedures based on PCR-targeting, according to [19]. To construct the BCY20 strain, the Yih1-VN-tagged strain (VN_3198) was transformed with a PCR product generated by the amplification of the pFA6a-VC-His3MX6 module [19], with the following target-gene specific primers containing nucleotides downstream and upstream of the translation termination codon of *CDC28*: BC604 (CGCCAGAAGAGCAGCCATCCACCCTACTTCCAAGAATCAGGTCGACGGATCCCCGGGTT) and BC605 (CAGTAGCATTTG TAATATAATAGCGAAATAGATTATAATGCCGATGAATTCGAGCTCGTT). The construction of BCY21 strain followed the same procedures except that the strain E57 was used for transformation. The correct tagging was validated by immunoblot with antibodies against Cdc28. To construct the strain ESY10075aa, the *YIH1* ORF was replaced by the *Kan* ^*R*^ gene following published procedures [20].

The plasmid for expression of GST-Yih1 in *E*. *coli* was constructed by transferring a *BamH*I-*Sal*I fragment comprising the full-length sequence of *YIH1* cloned in pES191-H1 [13] into the same restriction sites in pGEX6-p3 (GE Healthcare) in frame with GST, generating plasmid pBE718. For construction of the plasmid expressing His_6_-Cdc28 in *E*. *coli*, the *CDC28* gene was PCR-amplified using the following primers: ESRC5 (CGCGGATCCGGGAATTCCGGTGGTGGTATGAGCGGTGAATTAGCAAATTAC) and ESRC6 (GCGGTCGACTCGAGCTATGATTCTTGGAAGTAGGGGTG). The PCR fragment was digested with *EcoR*I and *Xho*I and cloned into pETduet-1 (Novagen), generating plasmid RCB14. For expression of full-length IMPACT in N2a cells a *Bgl*II-*Bcl*I fragment comprising the complete IMPACT coding sequence (pBE610) [14] was transferred into the *Bgl*II site of pFLAG-CMV5a (pBF311) generating plasmid pBE626.

### Flow-cytometry analysis

For the analysis of the DNA content, yeast cells were grown in yeast extract, peptone, dextrose (YPD) or Synthetic Dextrose (SD) medium to an A_600_ of approximately 0.4–0.6. For overexpression of GST-Yih1 or GST alone, cells were grown to log phase in synthetic medium with 2% galactose. 1x10^6^ cells were fixed in 70% ethanol overnight at 4°C, harvested, resuspendend in 1 mL of 50 mM citrate buffer, pH 7.4 and sonicated for 10 seconds at setting 30% (1 sec on, 1 sec off) to disperse any potential clumps of cells. Cells were harvested, resuspendend in 1 mL of 50 mM citrate buffer containing 0.25 mg/mL RNAse-A (Sigma) and incubated for 2 hours at 50°C. Proteinase-K (Sigma) (1 mg) was added and cells were incubated for additional 2 hours. Cells were harvested and resuspended in 1 mL of 50 mM citrate buffer, pH 7.4, containing 16 μg/mL of PI (Propidium Iodide) (Sigma). Cells were incubated for 30 minutes at room temperature (RT), sonicated as above for 5 seconds and analyzed on a FACS Canto flow cytometer (BD Biosciences), acquisition of 1x10^4^ events. The proportion of cells in each cell cycle stage was assessed with Flowjo software (version 9.0.2, Tree Star Inc, San Carlo, CA), Watson-pragmatic model which fits the Gaussian DNA distribution curves to the stages of the cell cycle. Cell doublets were removed prior to analysis by plotting the area versus the width of FL-2 channel using the Flowjo software (version, 9.3.3, Tree Star inc, San Carlo, CA). For BiFC, cells were grown in SD medium to log-phase and washed in phosphate buffered saline (PBS). Live cells fluorescence levels were determined (2x10^4^ events) on a FACSCanto flow cytometer (BD Biosciences). Yeast strain RRY62a which should not have a BiFC signal showed intrinsic auto fluorescence similar to untagged strains, and was used to set the fluorescence baseline.

### Cell cycle time courses

To synchronize cells in G1, the mating pheromone alpha-factor (α-factor) (Sigma or Zymo-Research) was added to log-phase cultures grown to A_600_ of 0.4–0.6 (*MATa* yeast strains MSY-WT2 and MSY-Y2) to a final concentration of 15 μg/mL. Cells were incubated for 1 hour at 30°C at which time an additional dose of 7.5 μg/mL of α-factor was added and cells incubated for one more hour. Cells were monitored periodically by microscopy. Cells were collected by centrifugation, washed twice with pre-warmed medium and released into fresh pre-warmed medium at 30°C. Samples were collected at different time intervals and prepared accordingly. To synchronize cells in metaphase, two doses of nocodazole (Sigma) to a final concentration of 15 μg/mL each were added to log phase cultures, with a 1.5 hour interval. One and a half hours after the last dose, the cells were monitored by microscopy and released from the block as above. For time course experiments, yeast strains MSY-WT2 and MSY-Y2 were grown in YPD and samples were collected for DAPI (4′,6-diamidino-2-phenylindole dihydrochloride) staining and bud scoring by phase contrast microscopy. For experiments involving galactose induced expression of Yih1, yeast strain ESY-11b harboring a plasmid expressing GST-Yih1 was grown and released into fresh pre-warmed selective S medium containing 2% galactose as carbon source. Cells were collected for the preparation of whole cell extracts (WCEs) following the procedures described in [21], and for the assessment of DNA content by flow cytometry. Arrest and release assays shown herein were repeated at least three times.

### Fluorescence microscopy analyses

Between 1x10^7^ to 2x10^7^ cells grown to log phase in YPD were harvested and resuspended in buffer containing 40 mM KPO_4_ (pH 6.5) and 500 μM MgCl_2_. Cells were fixed with 3.7% formaldehyde and kept at 4°C for 4 hours. Fixed cells were washed two times with the above buffer and once in the same buffer containing 1.2 M sorbitol (sorbitol buffer). Cells were resuspended in 1 mL of the sorbitol buffer containing 0.25 mg of zymolyase 20T (Zymo-Research) and incubated at 30°C for 15 minutes. Cells were spun down (3 minutes 1500g), washed once with sorbitol buffer and immediately placed on ice. Cell suspensions were spotted on the wells of a Teflon-slide coated with 0.1% polylysine (Sigma) and incubated for 30 minutes at RT. The liquid was aspirated and cells were permeabilized with 0.05% Triton-X in PBS for 5 minutes, washed twice with PBS and blocked with 1% BSA in PBS for 30 minutes at RT. Cells were then incubated with the primary anti-tubulin antibodies (Invitrogen, 322500) at a 1:200 dilution in PBS, for 1 hour. Cells were washed three times with PBS and incubated with Alexa Fluor 488-conjugated anti-mouse IgG antibodies (Invitrogen), diluted 1:200 in PBS with 10 μg/mL DAPI for 1 hour at RT. Cells were washed four times with PBS. Cover slips were mounted with fluorescence mounting medium (Sigma) and sealed with nail polish. To stain the nuclear DNA of live yeasts, cells were grown in YPD. DAPI (Sigma) was added directly to the medium of logarithmically growing yeasts (A_600_ = 0.4) to the final concentration of 2.5 μg/mL. After 15 minutes cells were washed 3 X in PBS, mounted on a pad of 1% agarose in SD media and subjected to microscopy. For BiFC experiments, cells were grown to log phase in SD media. Cells were stained with DAPI and mounted as above. Fluorescence and phase contrast microscopy were performed using an Olympus BX51 epifluorescence microscope equipped with an Olympus DP71 CCD camera, using image Pro-Plus 6.2 software (Media Cybernetic) with a 100X oil immersion objective. For BiFC, image acquisition time was 300 to 450 ms, gain: 4, gamma: 1.9, offset: 131 in a standard fluorescein isothiocyanate green filter set.

### *In vitro* pull-down assay

Expression of recombinant GST-Yih1 was carried out in *Escherichia coli*—Rosetta (DE3) as follows. Cells were grown in LB medium containing 100 μg/mL ampicilin and 34 μg/mL cloramphenicol at 37°C until they have reached A_600_ of 0.2–0.4. Expression was then induced with 1 mM isopropyl-β-D-thiogalactopyranoside (IPTG) at 21°C overnight. After centrifugation, cells were lysed and the recombinant proteins (GST-Yih1 or GST alone) were purified under native conditions with glutathione beads (GE Healthcare). Proteins were eluted with glutathione (GE Healthcare) according to the manufacturer's instructions and dialyzed at 4°C against 30 mM HEPES, pH 7.4, 150 mM NaCl, and 1mM DTT, 10% glycerol for 8 hours at 4°C. Protein was purified as described in [14]. Protein concentration was determined with the Bradford assay [22]. In parallel, yeast whole cell extract (WCE) from a *yih1Δ* strain (MSY-Y2) was prepared as described [21], using GST pull-down breaking buffer (30 mM HEPES, pH 7.4, 200 mM NaCl, 1 mM EDTA, 1 mM DTT, 0.5% Nonidet P40 and EDTA-free protease and phosphatase inhibitor cocktail (Pierce)) and pre-cleared with protein-A Sepharose-beads. Purified GST-Yih1 (2, 4 and 6 μg) and GST alone (6 μg) were immobilized on glutathione-Sepharose beads (GE Healthcare) blocked with 0.5% BSA and subsequently incubated with 1 mg of yeast WCE for 1 hour at 4°C. Beads were washed five times with ice-cold GST pull-down breaking buffer and once with ice-cold GST pull-down breaking buffer without detergent. GST proteins were eluted with glutathione (GE Healthcare) according to the manufacturer´s instruction and precipitated with TCA. Proteins were resolved by SDS-PAGE and subjected to immunoblot analyzes. His_6_-Cdc28 was expressed in *E*. *coli* strain BL21 (DE3) (Novagen), as above, except that protein expression was carried out at 37°C for 2 hours. Yeast WCEs derived from wild type (strain MSY-WT2), expressing GST or GST-Yih1 under a galactose-inducible promoter (1 mg), were incubated with sepharose beads for 30 min at 4°C and harvested for 1 minute at 10,000 g at 4°C. The supernatant was then incubated with increasing concentrations of His_6_-Cdc28 (1, 2 or 4 μg) immobilized on Ni-NTA beads (QIAGEN). After incubation for 1 hour at 4°C, the resin was washed several times with ice-cold washing buffer (20 mM Imidazol, 50 mM Tris-HCl, pH 7.4, 150 mM NaCl, 1 Mm EDTA, 1mM DTT and EDTA-free protease and phosphatase inhibitor cocktail (Roche)). After the final wash the beads were resuspended in 40 μl 2X Laemmli's loading buffer and the supernatant was resolved by SDS-PAGE and subjected to immunoblot analyzes.

### *In vivo* pull-down assays

*In vivo* GST-pull-down assays from yeast WCEs prepared from asynchronous and synchronous cell cultures were performed as previously described [3, 12].

### Immunoblots

WCEs were prepared as described in [21]. Samples were resolved by SDS-PAGE and transferred to polyvinylidenefluoride (Millipore) or nitrocellulose membranes (GE Healthcare) as described in [23]. The following primary antibodies were used: anti-Cdc28 (Santa Cruz Biotechnology, Sc-6709), anti-CDK1 (Cell Signaling, #9112), anti-GST (Santa Cruz Biotechnology, Sc-459), anti-Histone-H3 (Abcam ab1791), anti-GAPDH (Sigma, G9545), anti-FLAG (M2) (Sigma, F3165), anti-Ser(P)^51^-eIF2α (Invitrogen, 44728G) and anti-Sui2 (eIF2α) serum produced in rabbit [24]. Membranes were incubated with horseradish peroxidase-conjugated goat anti-rabbit IgG, anti-mouse IgG (Santa Cruz Biotechnology), donkey anti-goat IgG (Pierce) or HRP-conjugated protein-A (GE Healthcare). Proteins were detected with ECL (GE Healthcare) using the digital imaging system LAS-4000 (GE Healthcare) or the Alliance 4.7 transilluminator (UVITEC Limited, Cambridge, UK).

### Immunoprecipitation of IMPACT-FLAG

N2a cells were grown in Dulbecco´s Modified Eagle´s medium (DMEM) containing 10% FCS and 1 mM sodium pyruvate as previously described [17]. Transfection with pBE626 or with the vector alone (pBF311) was performed with Lipofectamine 2000 (Life-Technologies) for 5 hours in Opti-MEM (Life-Technologies). Cells were lysed in ice-cold lysis buffer (50 mM Tris pH 7.4, 1 mM EDTA, 100 mM NaCl, 1% Triton X-100, EDTA-free protease and phosphatase inhibitor cocktail (Pierce)) as described [17]. The supernatant was pre-cleared with protein-A agarose beads (Ambion). The pre-cleared lysate was incubated with 15 μl of anti-FLAG (M2) affinity Gel (Sigma) for 1 hour at 4°C. Beads were washed four times with ice-cold lysis buffer and once with ice-cold lysis buffer without detergent. The complexes were eluted with the FLAG-peptide (Sigma) in lysis buffer without detergent according to the manufacturer instructions and the proteins were precipitated with 10% TCA. The complexes were analyzed by immunoblot.

## Results

### Deletion of *YIH1* leads to accumulation of cells in the G2/M phases of the cell cycle

**Fig 1:**
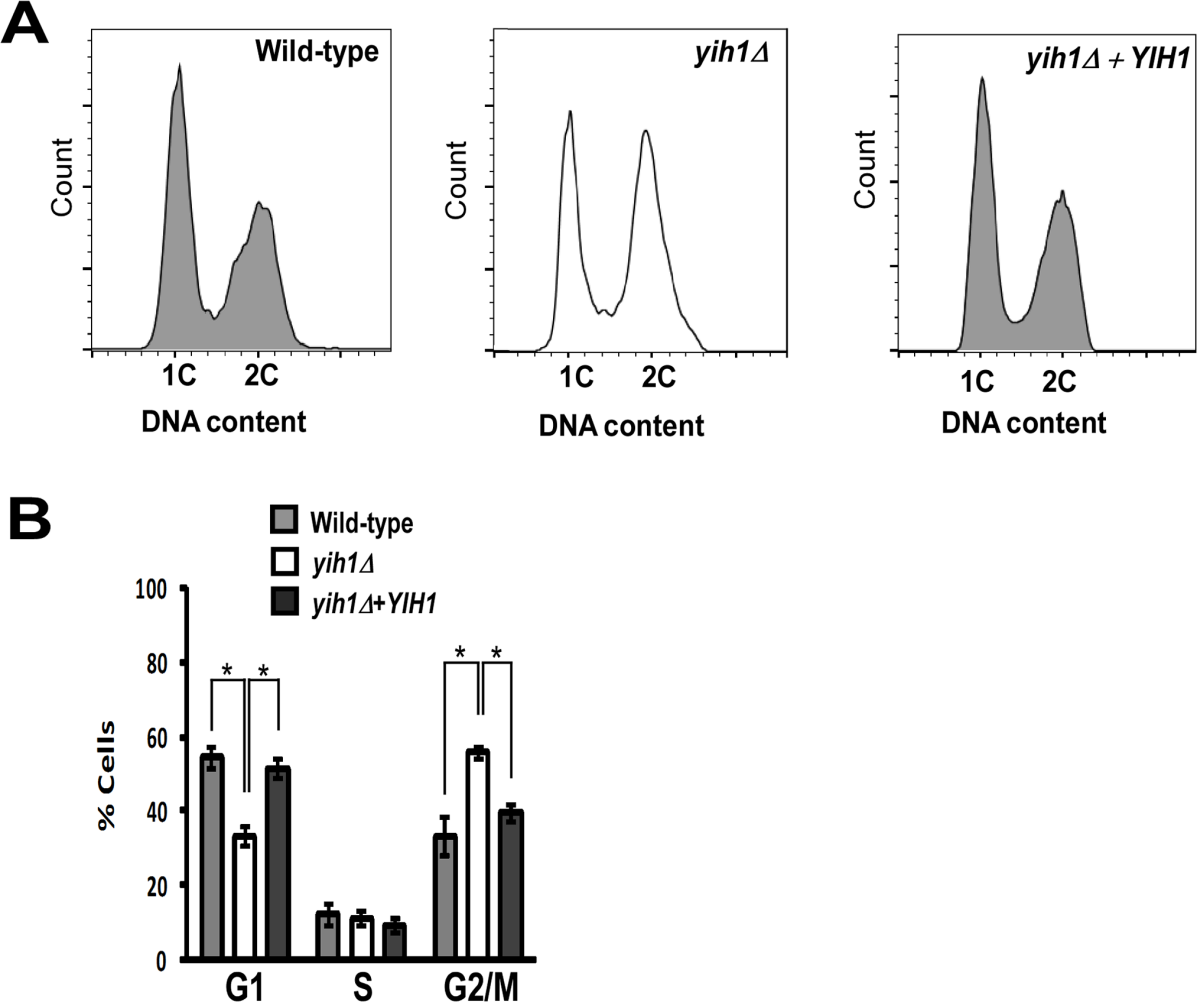
Deletion of *YIH1* leads to accumulation of cells with a 2C DNA content. **(A)** Representative histograms of DNA content of wild type (MSY-WT2), *yih1Δ* (MSY-Y2) and *yih1Δ*+*YIH1* (*YIH1* re-introduced into the chromosome of the *yih1Δ* strain, ESY-11b) cells grown in YPD to log phase, stained with PI, and measured by flow cytometry. **(B)** Quantification of the proportion of cells in G1 (1C DNA content), S or G2/M (2C), given as percentage of total cell population, determined from (A), using the flow cytometry gates with the cell-cycle tool (Watson model) of Flowjo software, 9.3.3 version. Data are presented as means ± S.E. (*error bars*) of three independent experiments.

In order to provide additional insights into the biological role of endogenous Yih1, we analyzed the cell cycle profile of asynchronous yeast cell cultures during exponential growth in rich media (YPD). We measured the DNA content by flow cytometry and found that, relative to the isogenic wild type strain, *YIH1* deletion leads to a significant increase in the percentage of cells with a 2C DNA content (WT: 33.4% ±5.1 vs *yih1Δ*: 55.6%±1.7), with a concomitant decrease in the percentage of cells with a 1C DNA content (WT: 54.3% ±2.6 vs *yih1Δ*: 33.2% ±5.4) (Fig 1). Reintegration of the *YIH1* gene into the chromosome of the *yih1Δ* mutant (*yih1Δ+YIH1*, strain ESY11B) fully restored the wild type cell cycle profile (Fig 1), indicating that the defect observed in *yih1Δ* mutants is due to the absence of Yih1.

**Fig 2:**
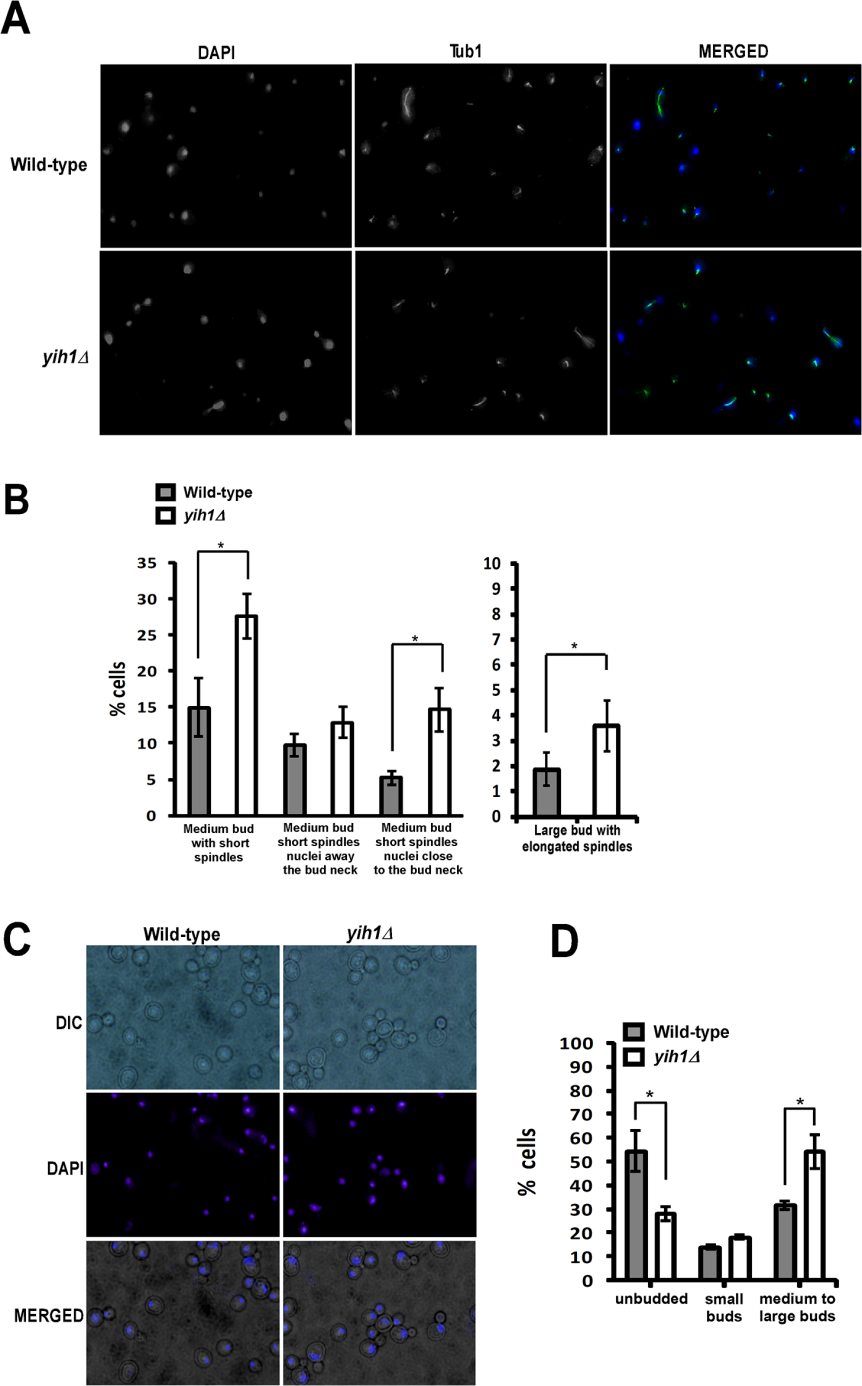
Deletion of *YIH1* leads to the accumulation of cells in the G2/M phases of the cell cycle. **(A)** Representative images of cells grown asynchronously to log phase in YPD medium, spheroplasted with zymolyase, permeabilized, fixed and stained with DAPI to visualize the nuclear DNA (left panels) and with anti-tubulin antibodies followed by Alexa-488 anti-mouse IgG to visualize spindles (middle panels). **(B)** Cells prepared as in A were counted and the percentage of cells with medium sized-buds and short spindles and the localization of the nucleus as indicated (left), or with large buds and elongated spindles (right), were determined. **(C)** Representative images of live exponentially growing yeast cells stained with DAPI. **(D)** Quantification of the percentage of cells in G1 (unbudded), S (cells with small buds) and G2/M (cells with medium to large buds) determined from (C). Over 450 cells were analyzed per experiment. Data represent the mean ± S.E. as error bars of three independent experiments for B and D. * *p*< 0.05; (Student´s *t* test).

Consistent with these results, analysis of cell morphology, nuclei staining with DAPI, and spindle morphology as detected by immunofluorescence with anti-tubulin antibodies, showed that in *yih1Δ* strains the proportion of medium size-budded cells with short spindles (indicative of cells in metaphase) was significantly higher than in the wild type strains (WT: 14.9±4.0 vs *yih1Δ*: 27.5±4.1) (Fig 2A and 2B), Interestingly, a higher proportion of *yih1Δ* cells had their nucleus located near the bud neck when compared with the wild type (WT: 5.2±0.89 vs *yih1Δ*: 14.7±43.0) (Fig 2A and 2B), indicating that cells lacking Yih1 do not accumulate during the S phase, but during the early stages of mitosis. In addition, the *yih1Δ* strain also presented a higher proportion of medium to large-budded cells with divided nucleus (54.0±7.2) relative to the wild type strain (31.0±3.1) (Fig 2C and 2D) and large-budded cells with elongated spindles (WT: 1.86±0.66 vs *yih1Δ*: 3.57±0.91) (Fig 2A and 2B), indicative of cells accumulating during anaphase, suggesting that the lack of Yih1 results in the accumulation of cells during the G2/M phases of the cell cycle.

**Fig 3:**
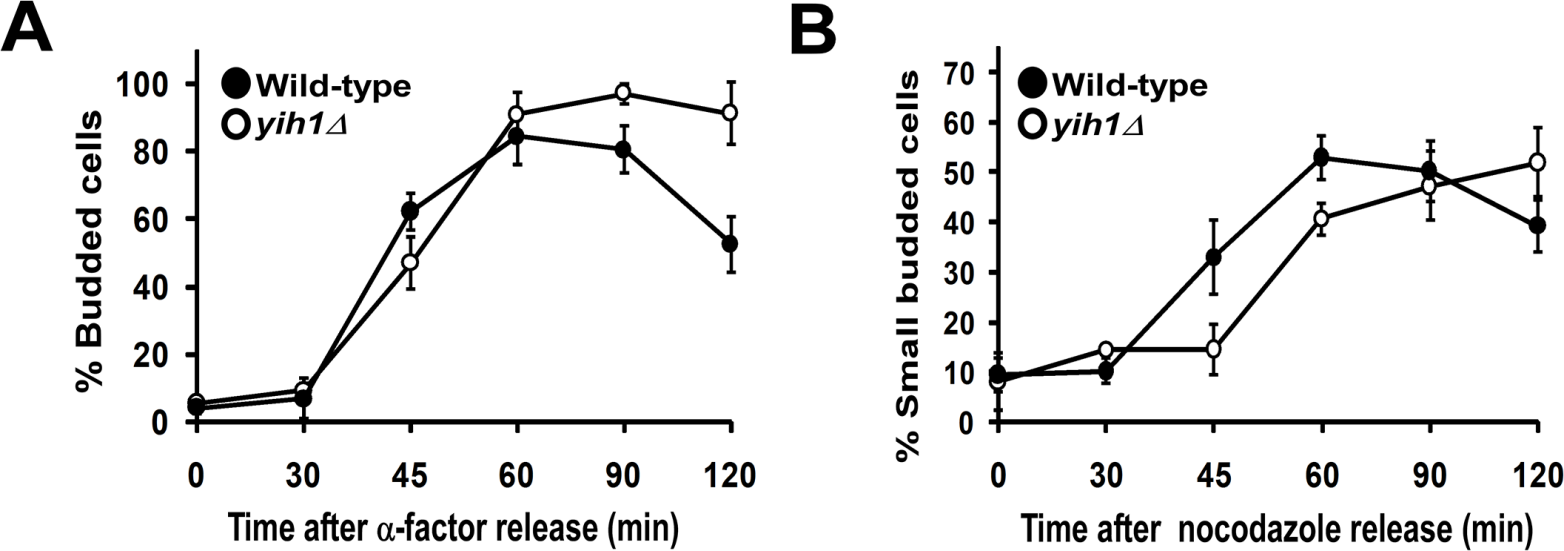
*yih1Δ* cells remain longer in the G2/M phases. **(A)** Exponentially growing wild type (MSY-WT2) and *yih1Δ* (MSY-Y2) cells were synchronized in G1 with α-factor and released into fresh YPD media. Samples were collected at the indicated time intervals and cells were fixed. The DNA was stained with DAPI. The percentage of budded cells (mean ± S.E. of three independent experiments) was determined. **(B)** Cells were synchronized in metaphase with nocodazole and released into fresh YPD media. Samples were taken and stained as in A. The percentage of small-budded cells (mean ± S.E. of three independent experiments) was determined. For each time point given in the graphs, more than 300 cells were analyzed.

To further characterize this cell cycle defect, the progression of wild type and *yih1Δ* cells through the cell cycle was compared. We first treated cells with the mating pheromone α-factor. In response to this pheromone, Far1 is activated by a signal transduction pathway to bind and inhibit Cln–Cdc28 kinases, resulting in a G1 cell cycle arrest [25, 26]. Cells were then released from G1 arrest by transferring to medium without α-factor, collected at the indicated time points and the proportion of budded cells was scored by microscopic inspection. At the time of α-factor release, at least 96% of cells were arrested in G1. Both wild type and *yih1Δ* cells presented similar budding kinetics up to the S phase (~30 minutes). However, after 60 minutes the *yih1Δ* strain showed a larger proportion of budding cells than the wild type, and an even larger difference at 120 minutes, suggesting that they remained longer in mitosis, leading to a consequent delay in cell cycle resumption when compared to wild type cells (Fig 3A). We also compared the cell cycle progression of cells arrested at metaphase with nocodazole (an inhibitor of microtubule polymerization) and then released into fresh medium. Accordingly, the completion of mitosis after release from nocodazole was delayed by at least 15 minutes in *yih1Δ* strains compared to wild type cells, as judged by the re-emergence of small buds, and this was followed by a delay in the progression of *yih1Δ* cells into G1 phase (Fig 3B). Thus, the combined morphological and flow cytometry analyses described above indicate that in the absence of Yih1, the cell cycle is delayed, probably in G2/M.

### The abnormal cell cycle phenotype of the *yih1Δ* mutant does not depend on Gcn2 or Gcn1

**Fig 4:**
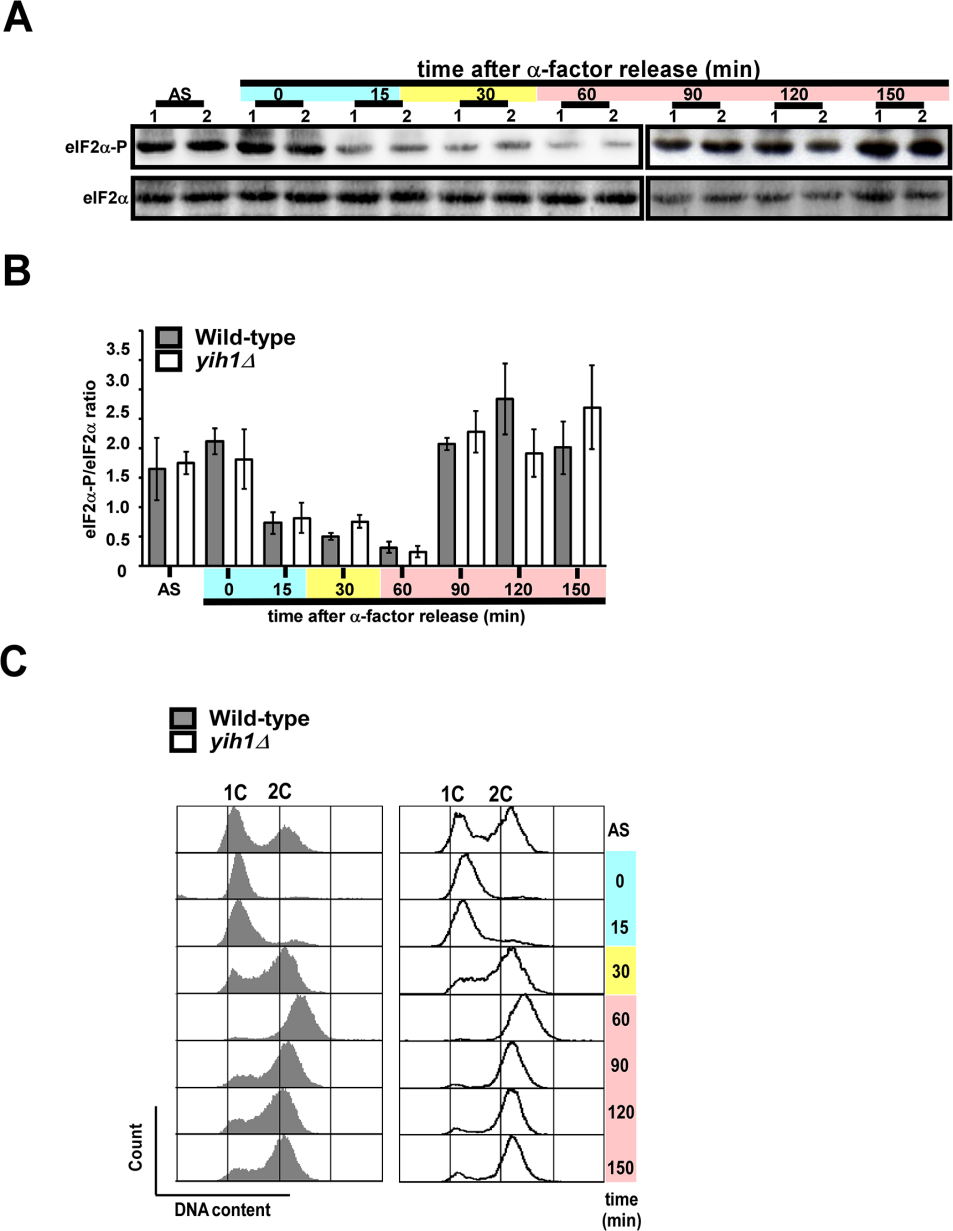
Phosphorylation of eIF2α along the cell cycle. Exponentially growing wild type (MSY-WT2) and *yih1Δ* (MSY-Y2) cells were arrested in G1 with α-factor and released into fresh SD media. Samples were collected at the indicated time intervals. **(A)** WCEs of these cultures and of an asynchronous culture (AS) were subjected to immunoblot analysis, using antibodies against eIF2α phosphorylated on Ser-51 (eIF2α-P) and against total eIF2α. **(B)** Immunoblot signals from three independent blots as shown in (A) were quantified using the NIH Image J software, the ratios between eIF2α-P and eIF2α were determined and the results were normalized using the value of the ratio of the asynchronous cells (AS); values represent means ± S.E.. **(C)** Representative histograms. Flow cytometry analysis of DNA content of the samples used in (A). Blue, yellow and pink shaded boxes indicate the estimated G1, S and G2/M cell cycle stages, respectively.

**Fig 5:**
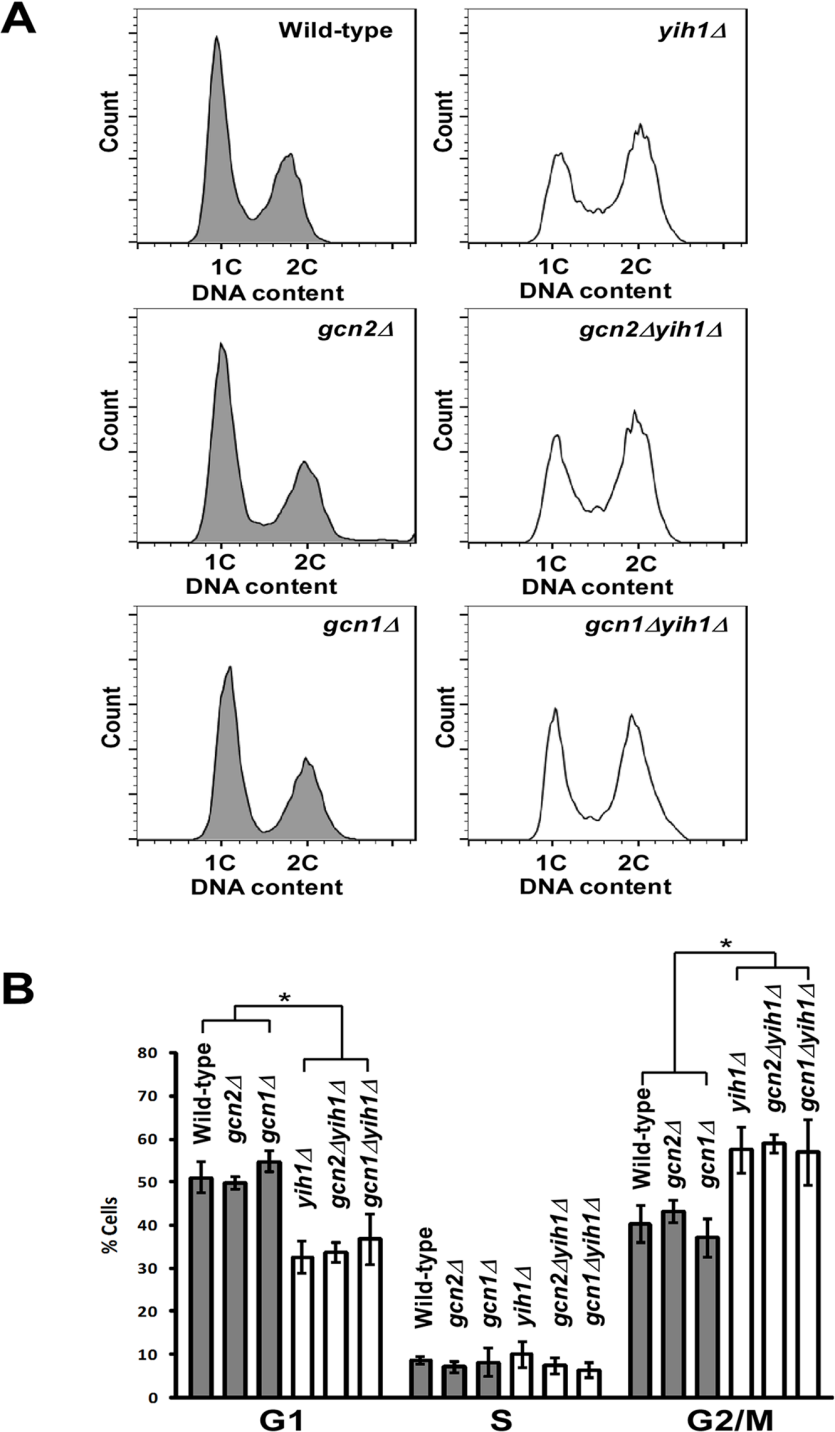
The abnormal cell cycle phenotype of *yih1Δ* cells does not depend on Gcn2 or Gcn1. **(A)** Representative histograms of DNA content of asynchronous cultures: wild type (H1511), and *yih1Δ* (ESY11001b), *gcn2Δ* (H2557) and *gcn1Δ* (H2556) single mutants, and *yih1Δ;gcn2Δ* (ESY10075aa) or *yih1Δ*;*gcn1Δ* (ESY10075aa) double mutants, grown to log-phase in YPD, stained with PI and analyzed by flow cytometry; the distribution of cells in G1 (1C), S or G2/M (2C) is shown. **(B)** The proportion of cells in G1 (1C), S or G2/M (2C), as determined from (A) is given as percentage of the total cell number, quantified using the flow cytometry gates depicted in A with the cell-cycle tool (Watson model) of Flowjo software, 9.3.3 version. Data are presented as means ± S.E. (*error bars*) of three independent experiments. * *p*< 0.05 (ANOVA).

We have previously shown that Yih1, when overexpressed, inhibits Gcn2 activation by competing with the latter for Gcn1 binding [13–15]. However, *YIH1* deletion does not result in increased basal or starvation-induced Gcn2 activation, as judged by eIF2α phosphorylation levels determined by immunoblots of whole cell extracts obtained from asynchronous cultures. Therefore, we reasoned that native Yih1 could be modulating the activity of Gcn2 in specific stages of the cell cycle, thus regulating the synthesis of proteins important for the progression of the cell cycle, which could account for the phenotype observed in yeast devoid of Yih1. Previous studies in mammalian cells indicated that the basal levels of eIF2α phosphorylation (eIF2α-P) increase during G2/M to inhibit global protein synthesis during mitosis [27, 28]. Here, we then monitored the basal levels of eIF2α-P in yeast arrested in G1 with α-factor and released into fresh medium without the pheromone. In agreement with the data obtained in mammals, we found that the levels of eIF2α-P abruptly declined during late G1 (~15 minutes), S phase (~30 minutes) and early mitosis (~60 minutes), but increased dramatically during the G2/M phase of the cell cycle (~90 to 120 minutes) (Fig 4A and 4B), as judged by flow cytometry analyses of the cellular DNA content of the same samples from which cell extracts were prepared for the analyses of eIF2α(P) levels (Fig 4C). Cells lacking Yih1 presented similar detectable levels of basal eIF2α-P in relation to wild type cells throughout the cell cycle, suggesting that Yih1 does not affect Gcn2 activity during the cell cycle; alternatively the experimental procedure may not be able to detect subtle changes in localized eIF2α phosphorylation. To further address whether Gcn2 or Gcn1 may be involved in the cell cycle progression defect observed in *yih1Δ* cells, the cell cycle profiles of mutants lacking both Yih1 and Gcn1 (*yih1Δ*;*gcn1Δ)* or Yih1 and Gcn2 (*yih1Δ*;*gcn2Δ)*, exponentially growing in rich media, were analyzed by flow cytometry. These double mutants were made in the H1511 genetic background. In agreement with the data obtained from yeast cells derived from the BY4741 genetic background (Fig 1A and 1B), deletion of *YIH1* in strains derived from the H1511 genetic background also resulted in an increased proportion of cells accumulating with a 2C DNA content when compared to their wild type counterpart (Fig 5A and 5B). The cell cycle profiles of the double deletion strains (*yih1Δ*;*gcn2Δ* or *yih1Δ*;*gcn2Δ)* did not significantly differ from that of the *yih1Δ* single mutant. Accordingly, the cell cycle distribution of single mutants *gcn1Δ* or *gcn2Δ* was essentially indistinguishable from the wild type strain (Fig 5A and 5B).

Altogether, these results indicate that neither Gcn1 nor Gcn2 are involved in the accumulation of cells with a G2/M DNA content observed in *yih1Δ* cultures. Moreover, it appears that Yih1 is not responsible for the reduction in eIF2α-P levels during the cell cycle. Importantly, these results suggest that Yih1 may have a Gcn2-independent function.

### Yih1 is in a complex with the cyclin-dependent kinase Cdc28

**Fig 6:**
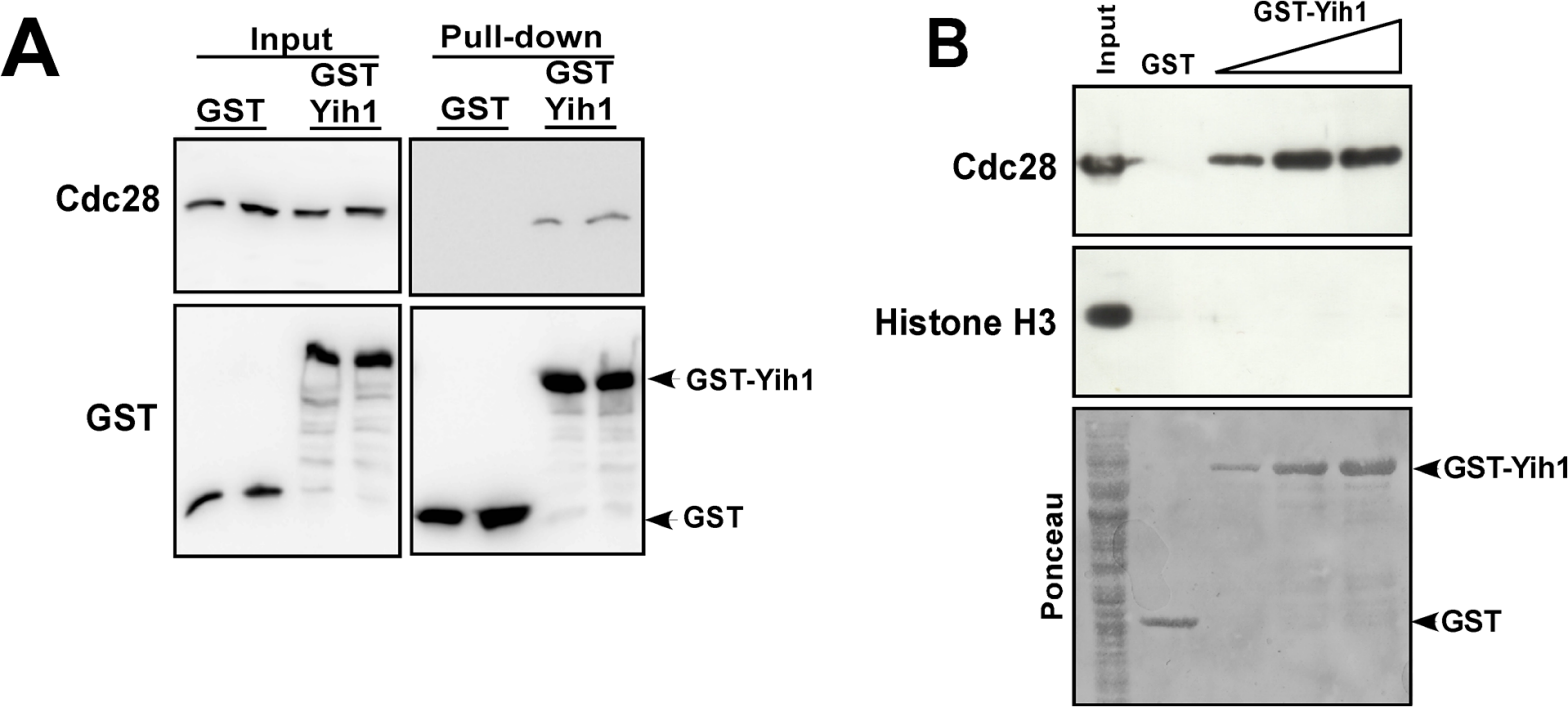
Cdc28 co-precipitates with GST-Yih1. **(A)** *In vivo* GST-pull-down assay. *yih1Δ* strains (MSY-Y2) expressing GST-Yih1 or GST alone from the galactose inducible promoter were grown to log-phase and harvested. Equal amounts of WCEs (2 mg) were subjected to glutathione-mediated GST pull-down assays. The precipitates (100% of the bound proteins – right-panel) and the input (1/100^th^ of the input – left panel) were assessed by immunoblot to detect the indicated proteins. **(B)** GST-Yih1 purified from *E*. *coli* co-precipitates endogenous Cdc28 from yeast WCEs. Full-length Yih1 fused to GST or GST alone were expressed in *E*. *coli*, purified, immobilized on glutathione-Sepharose beads and incubated with equal amounts of glutathione-Sepharose pre-cleared WCEs (1.25 mg) prepared from a *yih1Δ* strain (MSY-Y2). After extensive washes the precipitates (100% of the bound proteins) and the input (1/50^th^ of the input) were analyzed by immunoblot to detect the indicated proteins. The Ponceau staining of the membrane is shown (lower panel). One representative blot from two independent experiments performed in duplicate is shown.

In an attempt to identify additional Yih1 binding partners, we discovered that Yih1 interacted with one of the control proteins used in our in-house yeast-2-hybrid platform, the mammalian CDK1 protein [29]. The eukaryotic cell division cycle is mainly controlled by activation and inactivation of Cdks, protein kinases that phosphorylate numerous proteins. Cdk activity requires association with its positive regulatory subunits named cyclins, which also determine Cdk substrate specificity, and whose abundance fluctuates throughout the cell cycle. Cdk activity is also regulated by phosphorylation of its catalytic subunit and interaction of inhibitory proteins to the cyclin–Cdk complex. In *S*. *cerevisiae*, the cell cycle progression is driven by a single Cdk, Cdc28, the CDK1 orthologue [30, 31]. Thus, we aimed to investigate whether Yih1 interacts with Cdc28. We have established previously that overexpression of GST-tagged Yih1 in yeast cells followed by GST-mediated co-precipitation assays is a suitable approach for scoring the interaction of proteins with Yih1 *in vivo* [3, 13]. Thus, WCEs from *yih1Δ* strains expressing GST-Yih1 or GST alone were incubated with glutathione beads. The beads were extensively washed and the inputs and precipitates subjected to immunoblot analyses with antibodies against Cdc28 and GST. As shown in Fig 6A, Cdc28 co-precipitated specifically with GST-Yih1 but not with GST alone in independent experiments performed from two different yeast transformants. The same experiment was performed in a *gcn1Δ* strain and we found that the interaction of Yih1 with Cdc28 does not depend on Gcn1 *in vivo* (see below).

We then tested the ability of purified Yih1 to bind endogenous Cdc28 in yeast WCEs. Recombinant full-length Yih1 fused to GST or GST alone were expressed and purified from *E*. *coli*, immobilized on glutathione beads and incubated with WCEs obtained from *yih1Δ* cells. After extensive washings, the bound proteins were eluted and analyzed by immunoblot. Cdc28 present in the yeast extract was readily co-precipitated with increasing concentrations of added GST-Yih1 but not with GST-alone. Histone-H3, a protein not known to interact with Yih1, was used as negative control. Histone-H3 is a highly abundant basic protein, while Yih1 is an acidic protein that also occurs in the nucleus [32, 33]. Yet, histone-H3 did not co-precipitate with GST-Yih1, suggesting that the Yih1-Cdc28 association was specific (Fig 6B).

Next we analyzed whether recombinant purified Cdc28 can bind Yih1 present in yeast cell extracts. WCEs generated from *yih1Δ* cells expressing either GST-Yih1 or GST-alone from the galactose inducible promoter were incubated with recombinant His_6_-Cdc28 purified from *E*. *coli*, immobilized on nickel resin. After extensive washings, the pellets were analyzed by immunoblot. We found that Cdc28 does not precipitate GST-Yih1 from cell extracts (S1 File), suggesting that the interaction of Yih1 with Cdc28 may be potentially dependent on post translational modifications in Cdc28, or on Cdc28 activity (and associated with specific cyclins). It should be noted that recombinant Cdc28 proteins are functional [34–36] (see discussion).

Since Yih1 was overproduced in the experiments above, we then investigated whether native Yih1 interacts with Cdc28 under physiological conditions. Despite intensive efforts, the interaction between native Yih1 and native Cdc28 could not be detected by immunoprecipitation of Yih1 or Cdc28 (data not shown). The expression of Yih1 is relatively low in the cell (~3030 molecules per cell) [32] and therefore it is possible that Yih1 levels may be below the detection range of immunoblots.

**Fig 7:**
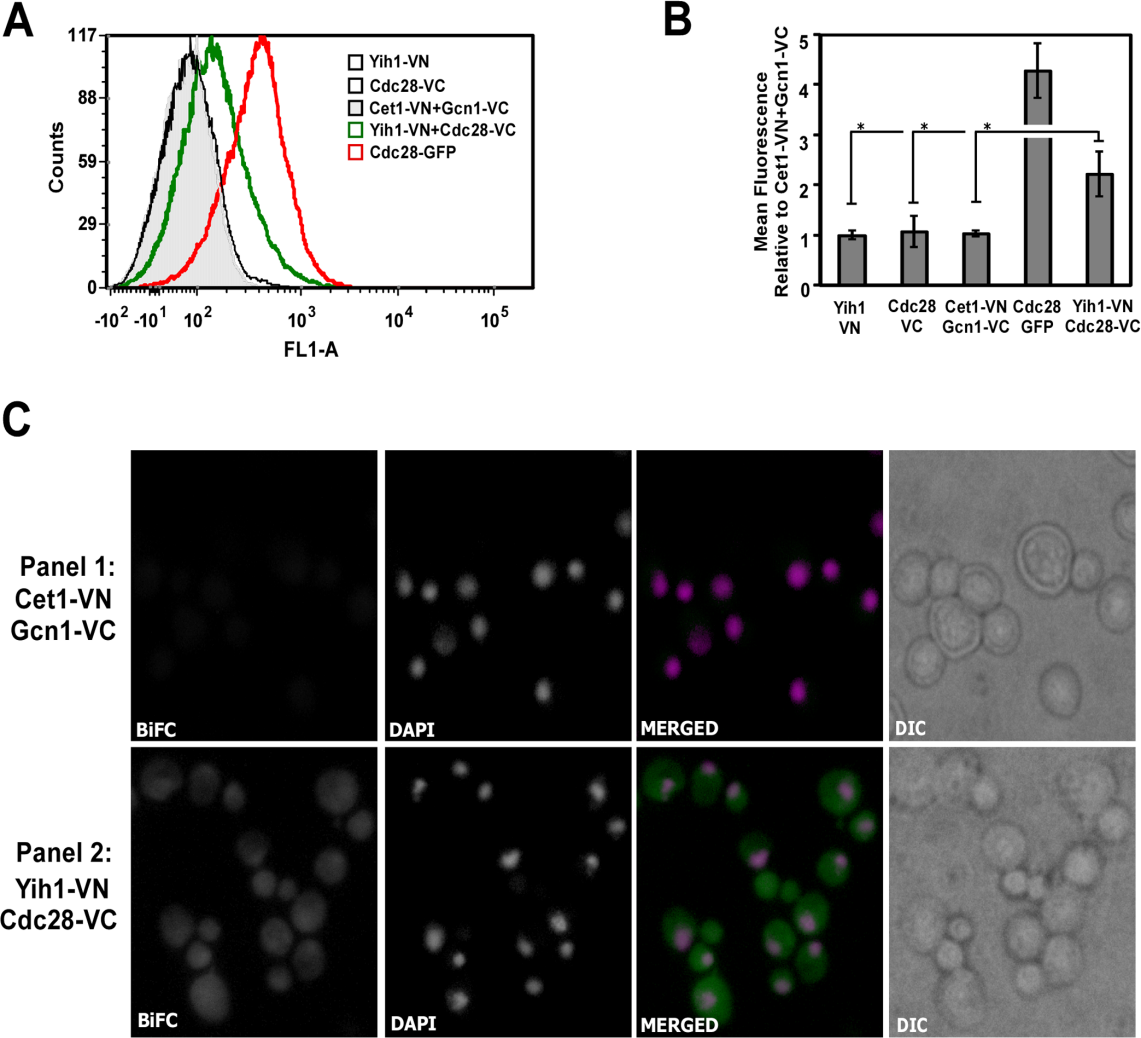
Endogenous Yih1 physically interacts with Cdc28. Visualization of Yih1-Cdc28 interaction by BiFC analysis. **(A)** Representative flow cytometry histograms of live cells grown in SD medium to log phase. 2x10^4^ cells were analyzed per acquisition using the FL1-A channel. The strain co-expressing the protein Cet1 tagged with VN and the protein Gcn1 tagged with VC (Cet1-VN+Gcn1-VC) was used to define the basal fluorescence signal (gray filled histogram). The strain co-expressing Yih1-VN and Cdc28-VC (BCY20) showed increased signal intensity, indicative of a positive BiFC signal (green unfilled histogram). Strains expressing Yih1-VN (VN_3198) or Cdc28-VC (BCY21) alone were negative (black unfilled histograms). A strain expressing Cdc28 fused to GFP (YBL160W) was used as a positive control (red unfilled histogram). **(B)** The mean fluorescence intensities of each cell population analyzed in (A) were determined using the FlowJo software, version 9.3.3. The bar graph shows the mean fluorescence intensities of the analyzed strains relative to the values measured in the strain Cet1-VN+Gcn1-VC, which was set to 1. Data represent mean ±SE as error bars of three independent experiments performed in duplicate. * *p* < 0.05; (Student´s *t* test). **(C)** Representative fluorescence images of live cells grown as in (A). The strain co-expressing Cet1-VN+Gcn1-VC (RRY62a) is shown in panel 1. The strain co-expressing Yih1-VN and Cdc28-VC (BCY20) is shown in panel 2. In the merged images, nuclear DNA staining with DAPI is shown in magenta, the BiFC signal is shown in green, and the colocalization of DNA with the BiFC signal from Yih1-Cdc28 is shown in white, using the software Image J. DIC is also displayed. Images for strains expressing Yih1 (VN_3198) or Cdc28 (BCY21) alone are provided in S2 File.

We then employed the Bimolecular Fluorescence Complementation (BiFC) assay, which provides information both on the presence of a physical interaction and on its subcellular localization. BiFC is based on the recovery of fluorescence after the two non-fluorescent halves of a yellow fluorescent protein variant (Venus), fused to two different binding partners, are brought together during interaction events [19]. The tagging of Cdc28 with a fragment of Venus protein and its interaction with a specific binding partner through fluorescent protein-fragment complementation assays has been previously reported [37], indicating that Cdc28 tagging for BiFC is a feasible approach for determining its interaction with Yih1 *in vivo*. We then created a yeast strain expressing the N-terminal half of Venus (VN) fused to the C-terminal end of Yih1 (Yih1-VN) and the C-terminal half of Venus fused to the C-terminal end of Cdc28 (Cdc28-VC). The fusions were made in the chromosomal copies of the *YIH1* and *CDC28* genes. Strains expressing only Yih1-VN or Cdc28-VC were used as controls. The detection of subtle fluorescence emission, as may be the case for fluorescence signals coming from protein-protein interaction episodes, potentially taking place in dynamic processes during the cell cycle, can be difficult. For this reason, we first monitored the association between Yih1 and Cdc28 in live cells by flow cytometry, which is highly sensitive and quantitative. We also included in this analysis a yeast strain expressing GFP fused to Cdc28, as an indicator of the relative signal strength for the BiFC. Cells growing exponentially in SD media were analyzed for fluorescence emission in FL1 (fluorescent green light). A BiFC signal was clearly detected when Yih1-VN and Cdc28-VC were co-expressed (Fig 7A, green line histogram). The BiFC signal was not detected in cells expressing either the VN-tagged Yih1 or the VC-tagged Cdc28 alone (Fig 7A, black line histogram). Moreover, to rule out the possibility that the positive BiFC signal was due to random interactions between the VN and the VC fragments within the cell, we analyzed a strain co-expressing two proteins not known to interact with each other, namely, Cet1, which was tagged with the VN fragment at its N-terminal end, and Gcn1, which was C-terminally tagged with the VC fragment. The resulting strain showed weak fluorescence signals comparable to those of other controls (Fig 7A, light gray filled histogram). Quantification of the mean fluorescence intensity (MFI) of each strain relative to the negative control (Cet1-VN-Gcn1-VC) is depicted in the graph shown in Fig 7B. We therefore concluded that the positive BiFC signal detected in the cells co-expressing Yih1-VN and Cdc28-VC originates from the proximity between the two halves of the Venus protein mediated by the interaction of Yih1 and Cdc28 *in vivo*.

In agreement with the flow cytometry analyses, the BiFC signal was also detected by fluorescence microscopy in cells co-expressing Yih1-VN and Cdc28-VC (Fig 7C, panel 2). Strains expressing two tagged proteins that do not bind to each other (Fig 7C, panel 1) or expressing only one of the tagged protein versions (Panels 1 and 2 in S2 File) presented only a very weak signal, if at all. The Yih1-Cdc28 BiFC signal was detected in the cytoplasm and in the nucleus, where both proteins were reported to be localized when fused to fluorescent tags [32, 33]. Taken together, these data give further support to the notion that Yih1 and Cdc28 proteins physically interact. Importantly, this assay provided evidence that this complex occurs under normal physiological conditions, in live yeast cells.

### Yih1 preferentially interacts with active Cdc28 complexes

**Fig 8:**
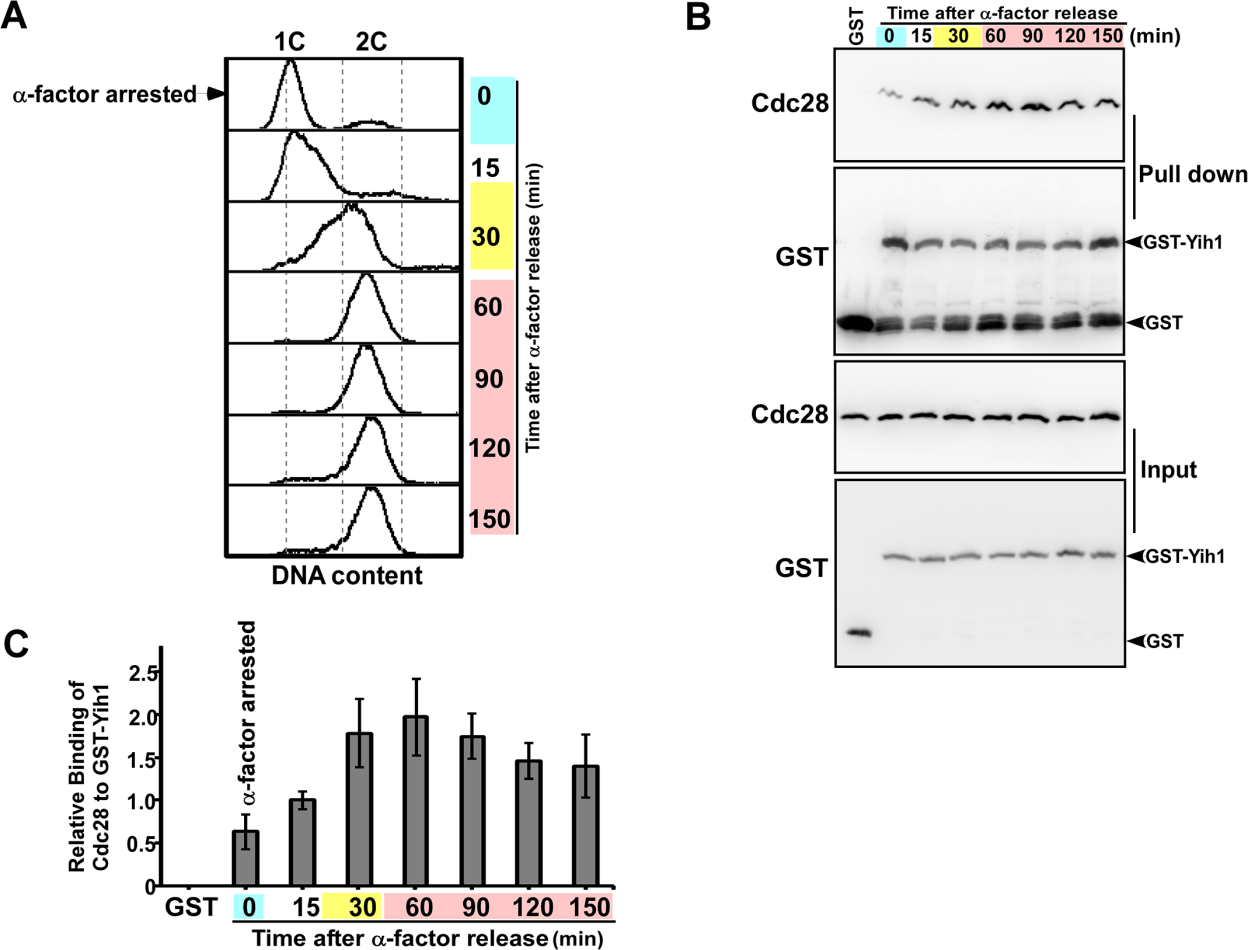
Evidence that GST-Yih1 preferentially binds to the Cdc28 active complex. *yih1Δ* cells (MSY-Y2) expressing Yih1 fused to GST from a galactose-inducible promoter were grown to log-phase in S medium containing galactose as carbon source (SGal). Cells were synchronized with α-factor, released into fresh SD media and samples were collected at the indicated times. **(A)** Representative histograms of DNA content (PI staining) of arrested (G1 – time 0) and released cells measured by flow cytometry. The distribution of cells in G1 (1C), S and G2/M (2C), analyzed with the Flowjo software, 9.3.3 version is shown. **(B)** Representative immunoblot of *in vivo* GST-pull-down assays. The collected cells were promptly harvested and equal amounts of proteins (1 mg) were subjected to glutathione-mediated pull-down assays. All the precipitated material (upper-panels) and 2% of the input (lower-panels) were subjected to immunoblots to detect GST proteins and Cdc28. As a negative control, GST alone was expressed in asynchronous *yih1Δ* cultures, pulled-down and analyzed as above. **(C)** The relative amount of Cdc28 bound to GST-Yih1 was determined with data from B using the NIH image J software. The amount of precipitated Cdc28 was normalized to the levels of precipitated GST-Yih1. Blue, yellow and pink shaded boxes indicate the estimated G1, S and G2/M cell cycle stages, respectively. Data represent mean ±S.E. of three independent experiments.

Having clearly established above that Yih1 is in a complex with Cdc28, a protein that is regulated in a timely manner throughout the cell cycle and is required for its normal progression, we then asked whether the Yih1-Cdc28 interaction is cell cycle-dependent. We analyzed the interaction of GST-Yih1 with Cdc28 along the cell cycle. A culture of *yih1Δ* mutant cells expressing GST-Yih1 from a galactose inducible promoter was grown in synthetic medium containing galactose, synchronized in G1 with α-factor and then released into fresh medium. Cell samples were collected at the indicated time points, WCEs prepared and used for glutathione pull-down and immunoblot assays as described above. Aliquots of all samples were analyzed in parallel for DNA content by flow cytometry to ascertain the cell cycle phase of the samples (Fig 8A). We found that the interaction between Yih1 and Cdc28 was at a minimum when cells were arrested in G1, and gradually increased upon release from G1 arrest. Yih1-Cdc28 interaction levels increased around S and M phases (30 and 60 minutes) and decreased again in the last two time points analyzed (120 and 150 minutes) (Fig 8C). Using the invariant expression of Cdc28 as reference [38], we found that GST-Yih1 protein levels remained constant throughout the cell cycle. Quantification of the amount of precipitated Cdc28 relative to GST-Yih1 indicated a two to three-fold increase in Yih1-Cdc28 interaction during S-G2/M phases (30, 60 and 90 minutes after release) relative to cells arrested in G1 with the α-factor (Fig 8C). These findings suggest that GST-Yih1 preferentially binds to active Cdc28 complexes involved in the S-G2/M phases of the cell cycle.

We also investigated whether the BiFC signal increases during the S-G2/M phases as would be predicted from our pull-down assays shown in Fig 8; however we did not see significantly increased fluorescence in budded cells compared to unbudded cells. This may be explained by the fact that the formation of a BiFC complex is not necessarily reversible [39], limiting the analysis of transient interactions occurring in dynamic processes such as the cell cycle.

### Mapping the interacting site on Yih1

**Fig 9:**
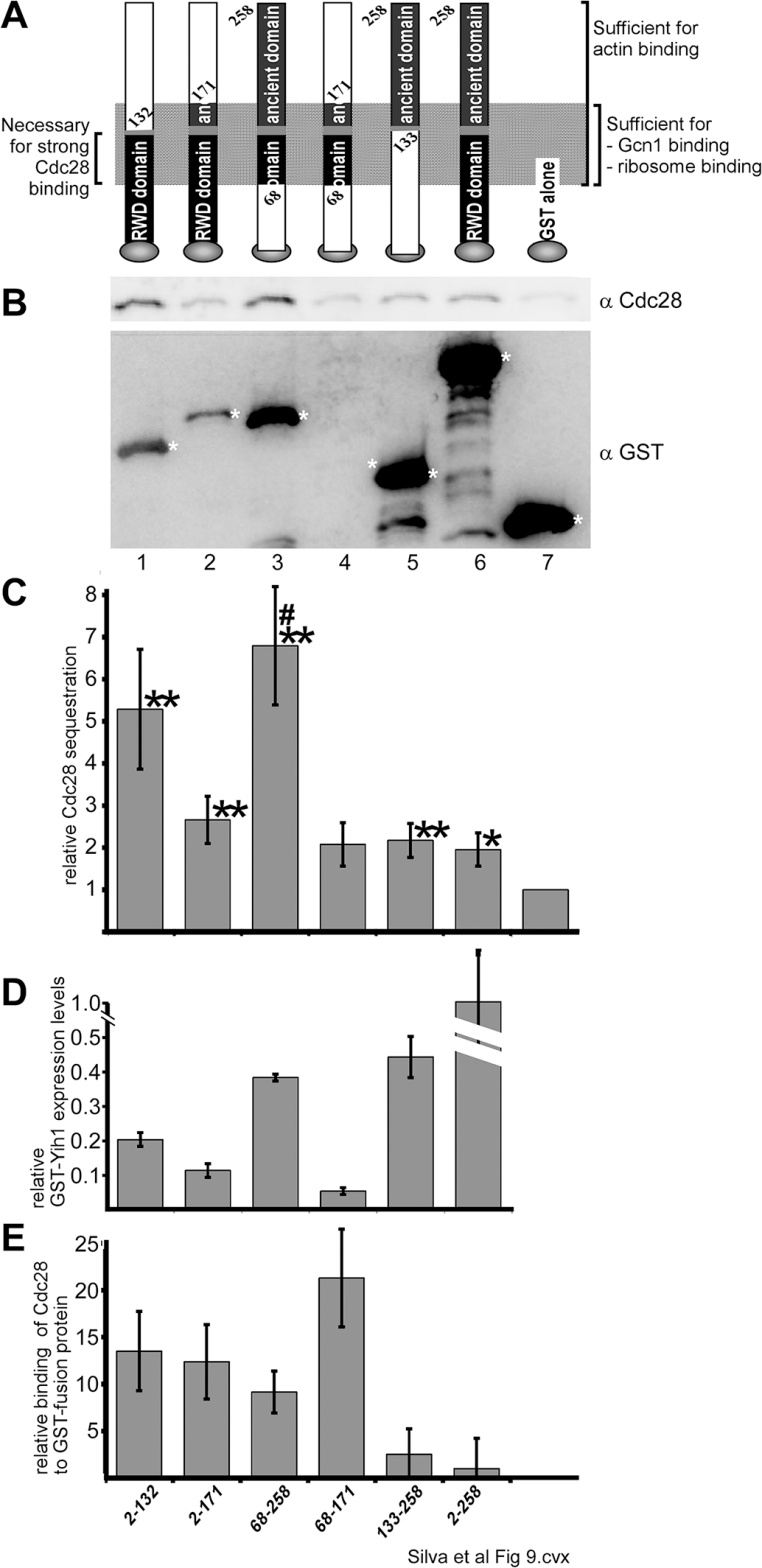
Cdc28 binding to Yih1 fragments. **(A)** Schematics of the GST-tagged Yih1 fragments used in this study. The N-terminal RWD domain and the C-terminal ancient domain are indicated, as well as the Yih1 region sufficient for actin, Gcn1, and ribosome binding (modified from [3]**)**. On the left, the Yih1 region encompassing the major Cdc28 binding determinant, as found in this study is shown. The schematic is not to scale. **(B)** Identical amounts of total protein from WCEs of the *gcn1Δ* strain H2556 overexpressing the indicated GST-tagged Yih1 fragments, were subjected to GST-mediated pull-down assays as described earlier [3]. The precipitated material was subjected to SDS-PAGE and immunoblot using antibodies against Cdc28 and GST. The assay was conducted at least six times, and a representative result is shown. * indicates the location of the respective GST-fusion protein in the blot. **(C)** The amount of Cdc28 sequestered in B was determined by quantifying the signal intensity of the Cdc28 signals from at least six independent results, using the NIH Image J software. The values are shown relative to those found for whole cell extracts containing GST alone. **(D)** The overexpression levels of the Yih1 fragments are shown relative to the expression level of full length GST-Yih1. Modified from [3] **(E)** The relative binding strength of GST-fusion proteins to Cdc28 was calculated by dividing the relative amount of Cdc28 sequestration in C by the relative expression levels of the respective GST-fusion proteins in D. The values are shown relative to that of GST-Yih1.

We next aimed to map the region of Yih1 required for Cdc28 binding. We performed pull-down experiments using a set of Yih1 fragments fused to GST and expressed from a plasmid and a galactose-inducible promoter in a *gcn1Δ* strain (Fig 9A) [3]. It has already been established previously that the efficiency of glutathione-mediated precipitation of these GST-Yih1 fragments is similar, but that they are not equally well overexpressed, and this was taken into consideration when interpreting the results, as done previously [3, 18]. Briefly, we determined the amount of Cdc28 sequestered by the GST-fusions by quantifying the amount of precipitated Cdc28, and the values were plotted in a bar graph relative to the value found for the WCEs expressing GST alone (Fig 9B and 9C). The relative binding strength between the Yih1 fragments and Cdc28 was determined by dividing the relative amount of sequestered Cdc28 (Fig 9C) by the relative expression level of the respective GST fusion protein (Fig 9D), and the values were normalized against that of full-length GST-Yih1[2–258] (Fig 9E).

We found that GST-Yih1[2–132], GST-Yih1[2–171], GST-Yih1[68–258], or GST-Yih1[133–258] sequestered more Cdc28 than GST alone, as found for full-length GST-Yih1 (Fig 9C), suggesting that these fragments contain the Cdc28 binding activity. The amount of GST-Yih1[68–171] precipitation was too low to be detected in westerns and yet it co-precipitated Cdc28 more than GST alone (Fig 9B, lane 4 vs 7, Fig 9C). From all Yih1 fragments, GST-Yih1[2–132], GST-Yih1[2–171], and GST-Yih1[68–258] bound Cdc28 the strongest (Fig 9E), suggesting that amino acids 68–132 contain the major Cdc28 binding determinant. Interestingly, these Yih1 fragments bind Cdc28 stronger than GST-Yih1, suggesting that the Yih1 N- and C-terminus negatively affect Yih1-Cdc28 interaction. Consistent with this idea, it seems that a Yih1 fragment lacking both termini—GST-Yih1[68–171]—bound Cdc28 even stronger than fragments lacking one or the other terminus (Fig 9E). GST-Yih1[133–258] appeared to bind Cdc28 at least as strong as GST-Yih1 (Fig 9E), suggesting that either the Cdc28 binding activity goes beyond amino acid 132, or that the ancient domain harbors an additional separate Cdc28 binding site. Together these results indicate that the major Cdc28 binding determinant in Yih1 overlaps with the Yih1 regions sufficient for binding actin, Gcn1, and ribosomes, and this binding determinant encompasses the predicted flexible linker region in Yih1 (Fig 9A) [3, 18].

### Effect of mutations in the Yih1 RWD domain on its interaction with Cdc28 and on the cell cycle

The RWD domain of Yih1 was previously modeled on the mouse Gcn2 RWD structure and revealed conserved residues in helix H2 and H3 that are possibly exposed to the solvent and thus may act as docking sites for Yih1 binding partners [3]. We have described that alanine substitutions of residues Asp-102 and Glu-106 in helix H3 within the RWD domain decreased the binding of GST-Yih1 (GST-YIH1*H3) to Gcn1 but not to actin [3]. Accordingly, this mutant protein was no longer able to inhibit Gcn2 when overexpressed. On the other hand, Yih1 with alanine substitutions of Glu-87 and Asp-90 in the helix H2 within the RWD domain (GST-Yih1*H2) interacts with its binding partners Gcn1 and actin stronger than the wild type Yih1, and overexpression of GST-Yih1*H2 resulted in a stronger inhibition of Gcn2 [3]. These substitutions in H2 may loosen up the structure of Yih1 so that its interactors may have an easier access to their binding sites; alternatively, these mutations may increase the affinity of the interactions.

**Fig 10:**
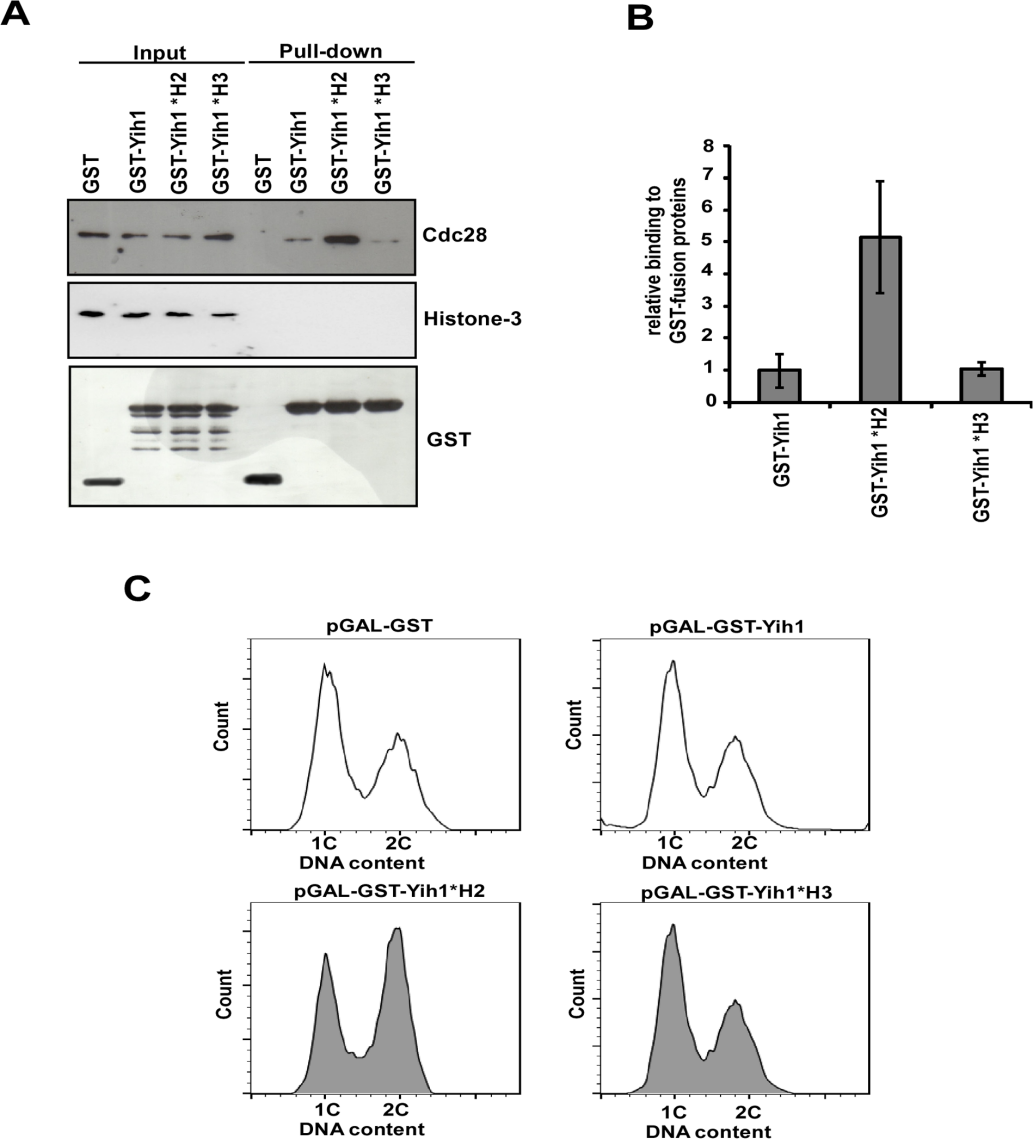
Effect of Yih1 RWD domain amino acid substitutions on Cdc28 binding. **(A)** *In vivo* GST-pull-down assays were performed on *yih1Δ* strains (MSY-Y2) expressing the GST-Yih1 fusion proteins as indicated, or GST alone, from a galactose inducible promoter. *H2 depicts E87A and D90A substitutions and *H3 depicts D102A E106A substitutions. Cells were grown to log-phase and harvested. Equal amounts of WCEs (2 mg) were subjected to glutathione-mediated GST pull-down assays. The precipitates (100% of the bound proteins – right) and the input (1/100^th^ of the input – left) were assessed by immunoblot to detect the indicated proteins. One representative blot from three independent experiments is shown. **(B)** Amount of endogenous Cdc28 bound to the GST-Yih1 fusion proteins was determined from (A) by dividing the signal intensity of Cdc28 by the precipitated amount of the respective GST-Yih1 fusion proteins. Data represent means and S.E. of three independent experiments. **(C)** Representative flow cytometry histograms showing the DNA content of wild type cells (MSY-WT2) expressing GST alone, GST-Yih1 (unfilled histograms), GST-Yih1*H2 or GST-Yih1*H3 (gray filled histograms) from a galactose inducible promoter. The distribution of cells in G1 (1C), S and G2/M (2C) is shown.

Here, we used these mutations in Yih1 to evaluate their effects on the binding to Cdc28 and on the cell cycle. Contrary to the decreased binding to Gcn1, and similarly to actin binding, GST-Yih1*H3 showed no significant defect in the association with Cdc28 in relation to the wild type GST-Yih1 (Fig 10A and 10B). GST-Yih1*H3 overexpression, as well as the overexpression of the wild type GST-Yih1, did not affect the cell cycle as evidenced by the DNA content of asynchronous cultures, determined by flow cytometry analysis (Fig 10C).

On the other hand, GST-Yih1*H2 co-precipitated far more Cdc28 than wild type GST-Yih1 (~5 fold increase) (Fig 10A and 10B), as was also the case for the binding to Gcn1 and actin [3]. No binding to histone H3 was detected, indicating that the increased interaction is specific. Interestingly, overexpression of GST-Yih1*H2 led to a phenotype similar to that observed in *yih1Δ* mutants, with cells accumulating with a 2C DNA content, as evidenced by flow cytometry analyses (Fig 10C). The stronger interaction of Yih1*H2 with Cdc28 together with its effect on the cell cycle provide further support to the view that Yih1 is involved in the modulation of the cell cycle during the G2/M stages of the cell cycle by means of Cdc28.

### Evolutionary conservation of Yih1-Cdc28 interaction

**Fig 11:**
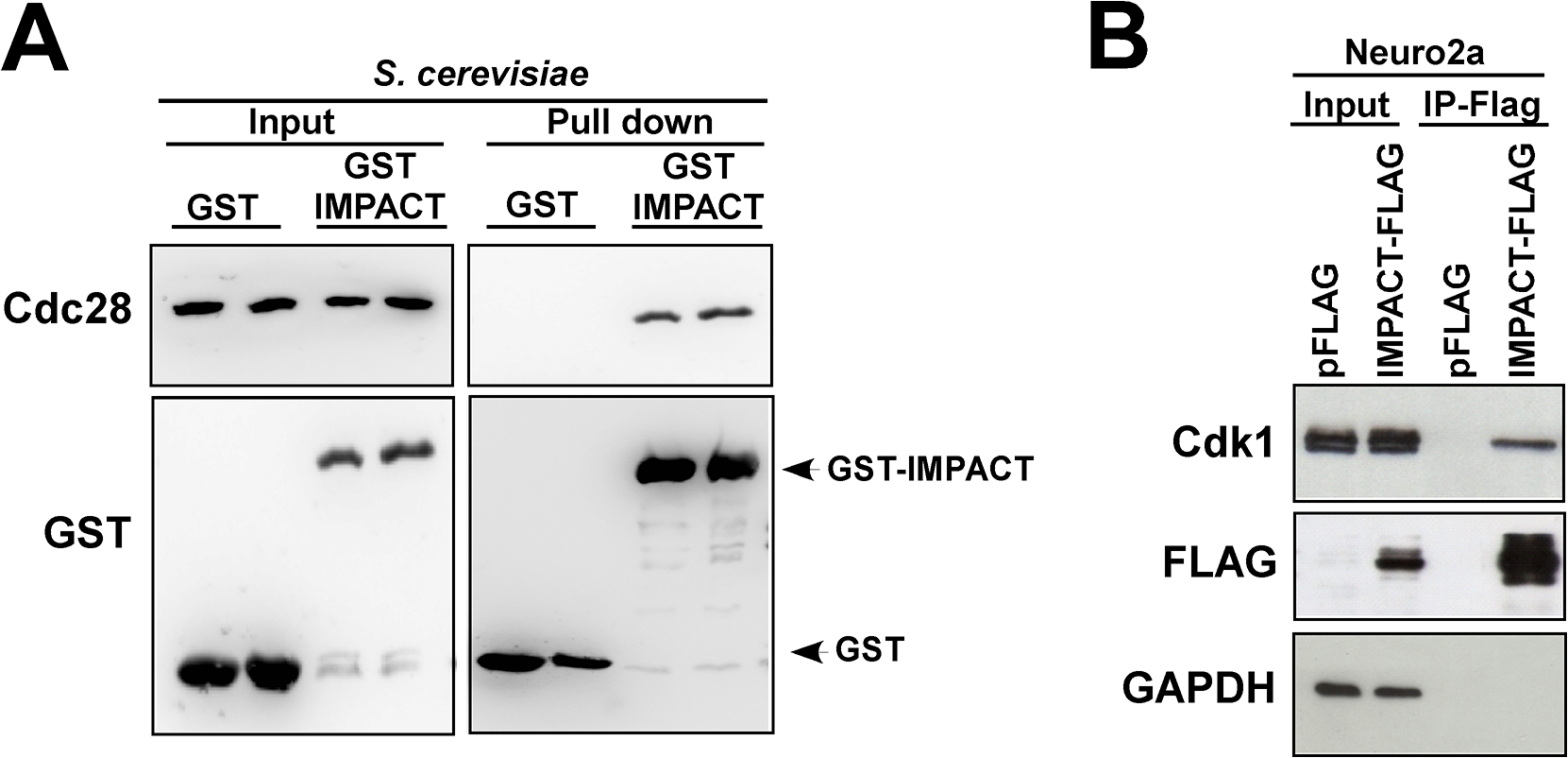
Mammalian IMPACT forms a complex with Cdc28 and CDK1. **(A)** Mammalian IMPACT expressed in yeast precipitates Cdc28. Two different transformants of *yih1Δ* strain (BY4741) expressing either GST-IMPACT or GST alone from a galactose inducible promoter were grown to log phase in SGal. WCEs were prepared and equivalent amounts of protein (1 mg) were subjected to GST-pull-down assays. The precipitated complexes were analyzed by immunoblot for the indicated proteins. The input lanes (left-panel) contained 4% of the WCEs used in the assay. **(B)** CDK1 co-precipitates with Flag-tagged IMPACT in N2a cells. Undifferentiated mouse N2a cells were transfected with a plasmid expressing IMPACT fused to Flag or with the vector alone (pFLAG). Cell lysates were cleared with protein-A agarose and subjected to immunoprecipitation with anti-Flag antibodies (M2-Flag-Resin). All the precipitated material and 1% of the input material were subjected to immunoblot to detect Flag-IMPACT, CDK1, and GAPDH as negative control.

IMPACT shares with Yih1 the ability to interact with yeast Gcn1, ribosome and actin [13, 14, 18], and when overexpressed in yeast, it inhibits the activity of Gcn2 to the same extent as Yih1 [14]. Cdk's are well conserved between *S*. *cerevisiae* and mammals. The mammalian CDK1 shares approximately 60–65% amino-acid similarity with its yeast homologue Cdc28 [40]. Accordingly, previous studies have shown that CDK1 can complement mutants of Cdc28 in *S*. *cerevisiae* [41, 42], highlighting the evolutionary conservation of the cell cycle control. The evolutionary conservation of Yih1/IMPACT prompted us to ask whether IMPACT also forms a complex with Cdc28. For this, *yih1Δ* strains expressing a plasmid-borne mouse IMPACT fused to GST, or GST alone, from a galactose-inducible promoter, were subjected to glutathione mediated co-precipitation studies as above. As shown in Fig 11A, Cdc28 specifically co-precipitated with GST-IMPACT but not with GST-alone. We next sought to determine whether IMPACT interacts with the mammalian orthologues of Cdc28. For this N2a cells were transfected with a plasmid for the expression of a Flag-tagged IMPACT or with the pFlag empty vector. The lysates were subjected to immunoprecipitation with anti-Flag antibodies coupled to protein-A beads. The immune-complexes were analyzed by immunoblot with antibodies against the Flag-tag and against mouse CDK1. High levels of CDK1 were found in the immunoprecipitate with IMPACT-Flag but not with the Flag alone, as shown in Fig 11B. As a negative control, the membranes were also probed for antibodies against GAPDH (Fig 11B). Thus, IMPACT interacts with Cdc28 in yeast and with its mammalian orthologue CDK1 in N2a cells. Since CDK3 has approximately the same molecular weight as CDK1 and the antibody used herein may cross-react with it, we cannot exclude the possibility that IMPACT interacts with CDK3. Taken together, these findings emphasize the evolutionary conservation of Yih1/IMPACT binding partners in different cellular contexts.

## Discussion

Here we described that endogenous Yih1 in the budding yeast functions as a modulator of the cell cycle. We showed by different approaches that the lack of Yih1 affects the timely progression through the cell cycle. Importantly, reintegration of *YIH1* into the chromosome rescues the cell cycle phenotype of *yih1Δ* mutants, indicating that the defects observed are directly related to Yih1. In *S*. *cerevisiae* it is difficult to distinguish between S, G2 and the start of M phase, one of the reasons being that the mitotic spindle already begins to form during early S-phase [43, 44]. Our analyses of cells released from α-factor arrest and of the number of medium budded-cells with short spindles in asynchronous cultures suggest that Yih1 is not involved in the completion of S-phase. All the data seem to suggest that Yih1 promotes the progression of cells through G2/M under normal growth conditions. Interestingly, our results indicate that cells lacking Yih1 delay during early and late stages of G2/M, as judged by the accumulation of medium size-budded cells with short spindles, indicative of premitotic and/or metaphasic cells, and of cells that had already undergone nuclear division, exhibiting an anaphase morphology, with elongated spindles. One possible interpretation of these results is that Yih1 may be required at different stages throughout G2/M. Despite the cell cycle delay, the growth rate of asynchronous cells lacking Yih1 in rich medium does not differ from that of wild type cells [13]. The α-factor arrest and release assays shown here indicated that both wild type and *yih1Δ* cells presented indistinguishable budding kinetics profiles from G1 to S phase, eliminating the possibility of compensatory shortening of the cell cycle stages that follow G2/M. Thus, we cannot exclude the possibility that those growth assays were not sensitive enough for detecting subtle differences.

Along the cell cycle of higher eukaryotes, cap-dependent translation peaks in G1 phase and decreases by 60 to 80% in mitosis mainly through the dephosphorylation of eIF4E-binding proteins (4EBPs). Only selected and specific mRNAs are translated in this phase to ensure accurate cell division. [45–47]. It has been previously shown in mammalian cells that the basal levels of eIF2α-P increase during G2/M, presumably as an additional mechanism to inhibit the global protein synthesis during mitosis [27, 28]. In yeast, our data suggest that the basal levels of eIF2α-P decreases during late G1, S and early G2/M stages of the cell cycle and increases again during later stages of mitosis in cells following release from α-factor arrest. It remains to be determined whether the variation in eIF2α-P levels is due to a cell cycle-dependent modulation of the activity of Gcn2 or whether it is due to cell-cycle dependent modulation in the activity/levels of the PP1 eIF2α-P phosphatase, Glc7, or in the ability of eIF2γ to target Glc7 to eIF2α-P [48, 49]. Nevertheless, in this work no difference was detected in the levels of eIF2α-P throughout the cell cycle between wild type and *yih1Δ* cells. However, this assay may not be able to detect subtle changes in localized eIF2α phosphorylation that may be modulated by Gcn2 and therefore be affected by Yih1. Thus, our previous suggestion that native Yih1 might act as a localized Gcn2 inhibitor to specifically ensure spatial or temporal regulation in a restricted manner cannot be ruled out [13].

In budding and fission yeast, Gcn2 is required to delay the G1/S transition upon DNA damage [6–8]. There are also precedents for the involvement of Gcn2 in the regulation of cell cycle progression in mammals under specific conditions [50, 51]. However, we have demonstrated that the cell cycle of asynchronous single mutants lacking Gcn1 or Gcn2 grown under normal conditions is not delayed, indicating that under normal, unstressed, conditions Gcn2 or Gcn1 do not affect the cell cycle. Double deletion strains *yih1Δ*,*gcn1Δ* and *yih1Δ*,*gcn2Δ* did not show any positive epistatic effect, suggesting that the cell cycle function of Yih1 is independent of its function as a Gcn2 regulator.

Supporting the notion that Yih1 is involved in the cell cycle, we found that Yih1 forms a complex with Cdc28. Cdc28 also co-precipitated with GST-Yih1 in WCEs of *gcn1Δ* cells indicating that the association with Cdc28 is not mediated via Gcn1. Importantly, we provided evidence that natively expressed Yih1 and Cdc28 form a complex *in vivo*, as determined by our BiFC assays, indicating that this complex occurs under physiological conditions. Cdc28 functions in both the cytoplasm and nucleus [30].

We have determined that recombinant Yih1 fused to GST purified from *E*. *coli* precipitates endogenous Cdc28 from yeast WCEs. Interestingly, however, recombinant Cdc28 fused to a His_6_-tag, purified from *E*. *coli*, did not precipitate overexpressed GST-Yih1 from WCEs. Given that several tagged versions of Cdc28 are functional [34–36], it is highly unlikely that the lack of interaction of recombinant His_6_-Cdc28 with ectopically expressed GST-Yih1 present in WCE is due to interference of the His_6_-tag. It is possible then that the interaction is dependent on a post-translational modification in Cdc28, or that the interaction is mediated by another protein that may not be sufficiently abundant in the WCE to allow the interaction of GST-Yih1 with the recombinant Cdc28 protein. Cdc28 exists both as a monomer with no catalytic activity and in association with its activating subunits, cyclins, and with several other positive and negative regulators. Since Cdc28 resides in a heterogeneous protein complex that is constantly changing throughout the cell cycle [52], it is possible that the association of Yih1 with Cdc28 is mediated by a cyclic, low abundance factor that would not be sufficiently available in a free form to associate with the His_6_-Cdc28 recombinant protein to mediate the interaction of His_6_-Cdc28 with endogenous Yih1.

Remarkably, the interaction between Yih1 and Cdc28 is weaker in extracts of α-factor mediated G1 arrested cells, when Cdc28 is inactive due to low concentrations of cyclins and the presence of cyclin dependent kinase inhibitors such as Far1 and Sic1 [31, 53]. Yih1-Cdc28 complexes gradually increase in abundance at late G1 (15 min after α-factor release) when the concentrations of cyclins rise and cdk inhibitors are degraded [52]. Cdc28-Yih1 interaction further increases during S (30 min) and G2/M (60–90 min), when the activity of Cdc28 is known to be high [52]. The interaction decreases slightly later in G2/M (120–150 min), when Cdc28 activity drops for cells to exit mitosis [53–55]. These observations suggest that Yih1 may interact with Cdc28 specific complexes along the cell cycle.

We cannot eliminate the possibility that α-factor-specific responses have an influence in the fluctuation of this interaction. Collectively, however, these results suggest that Yih1 forms a complex with active Cdc28, presumably bound to B-type (Clb) cyclins. Our attempts to detect cyclins co-precipitating with native Yih1 were not successful; however, we cannot exclude the possibility that this is because Yih1 is in low abundance and/or because commercially available cyclin antibodies are not very strong. Further investigations are required to determine to which Cdc28 complex Yih1 is associated with.

During G2/M Clb-Cdc28 complexes are targeted to different subcellular locations and phosphorylate a wide range of substrates involved in the switch from polarized to isotropic growth, in the assembly and orientation of the mitotic spindle, and in the activation of the mitotic exit network for cells to exit from mitosis [30]. Since the Yih1-Cdc28 interaction can be detected both in the nucleus and cytoplasm, and that deletion of *YIH1* delays the cell cycle progression in different points during G2/M, we consider it likely that Yih1 might be involved in the modulation of several different Cdc28 complexes during G2/M in both cellular locations. It is still not clear how Yih1 might function to positively modulate the cell cycle progression. One possibility is that Yih1 may somehow be necessary to keep the Clb-Cdc28 complexes functional. Loss of Yih1 may affect the activity of Cdc28 or even allow the association of specific cdk inhibitors to Clb-Cdc28 complexes. Alternatively, Yih1 might have a specific function in directing Cdc28 to one of its substrates or it might have a regulatory function in Cdc28-related or downstream pathways during G2/M. Supporting the view that the delayed G2/M phenotype of *yih1Δ* cells is modulated by Cdc28 complexes in budding yeast is our findings regarding the Yih1*H2 mutant containing amino acid substitutions in the RWD domain. GST-Yih1*H2 shows a stronger interaction with Cdc28 compared to wild type GST-Yih1, and its overexpression leads to a cell cycle phenotype similar to that observed in *yih1Δ* cells. We propose that the tighter binding of Yih1*H2 could stabilize Cdc28 active complexes, consequently preventing cells from exiting mitosis. This tight binding could also be hindering the access of Cdc28 to one of its substrates during G2/M or the access of specific kinases to phosphorylation sites on Cdc28, leading to the observed G2/M delay.

Yih1 does not seem to be a Cdc28 substrate because it lacks the S/T-P-x-K consensus sequence (where x is any amino acid) or the minimal consensus S/T-P sequence; although it is known that at least *in vitro* CDK1 can phosphorylate non-S/T-P sites [55]. In agreement with this, Yih1 has not been identified in different large scale studies aiming to find novel Cdc28 substrates [56, 57].

In contrast to yeast, mammalian cells possess several Cdks, which are regulated by multiple cyclins during each stage of the cell cycle. In mammals, the Cdc28 orthologue, CDK1, shuttles between the nucleus and the cytoplasm and specifically regulates the G2/M cell cycle transition with the aid of the cyclin B1 [58].

Here, we have shown that mouse IMPACT ectopically expressed in yeast co-precipitates Cdc28. In addition, CDK1 (or CDK3) co-precipitates with IMPACT in undifferentiated N2a cells, indicating that this interaction is evolutionary conserved, and reinforcing the notion that Yih1 and IMPACT are functional orthologues. IMPACT is a developmentally regulated protein highly expressed in neurons, which positively regulates neurite outgrowth induced by serum withdrawal in N2a cells. This phenotype seems to be only partially dependent on Gcn2. Interestingly, the levels of endogenous IMPACT increase upon neuronal differentiation [17], whereas the activity of Cdks declines [59, 60]. Therefore, it is tempting to speculate that IMPACT could be modulating the activity of Cdks by directly binding to it to allow cells to exit the cell cycle, thus triggering neuronal differentiation.

It is known that several proteins involved in the assembly of F-actin cables are substrates of Cdc28 [57, 61]. Consistent with this, it has been recently demonstrated that inhibition of Cdc28 activity reduces actin cable assembly rates and decreases the signal intensity of Alexa-568-phalloidin stained cables *in vivo* [62]. Interestingly, we have previously demonstrated that although the deletion of *YIH1* does not alter the morphology of the actin cytoskeleton, it reduces the proportion of cells stainable with rhodamine-phalloidin [13]. The basis for such reduction is unknown but it raises the intriguing possibility that the deletion of *YIH1* could be blocking the activity of Cdc28, consequently compromising the assembly of actin cables, accounting for the decreased levels of F-actin observed in yeast devoid of Yih1. Alternatively, the F-actin reduction observed in cells lacking Yih1 could compromise the function of Cdc28. Also, bud formation involves a dramatic rearrangement and polarization of the actin cytoskeleton prior to nuclear division. Actin disorganization triggered by different cues induces the morphogenesis checkpoint, which delays the cell cycle in G2/M until actin can repolarize and just then complete bud construction [63]. The cell cycle phenotype observed in *yih1Δ* cells could then be a result of actin disorganization. In view of the association of Yih1 with monomeric G-actin, it would be interesting to determine whether the actin cytoskeleton interferes with the association of Yih1 with Cdc28 and whether this competition is relevant to the modulation of the cell cycle. Since Cdc28 regulates the actin cytoskeleton and Yih1 also binds actin [3, 43], it is also possible that the Cdc28-Yih1 interaction is bridged through a common binding partner.

Our mapping of the Cdc28 binding determinants in Yih1 indicates that it involves the intrinsically disordered flexible linker region enriched in charged residues that connects the RWD and ancient domains in Yih1. In addition, our results indicate that the major Cdc28 binding determinant in Yih1 overlaps with the Yih1 regions sufficient for binding actin, Gcn1, and ribosomes. Interestingly, areas of structural disorder and low sequence conservation are commonly associated with docking sites for different regulatory proteins involved in different signaling pathways [64], including cell-cycle-controlled processes [65]. It remains to be determined whether Yih1 binds to a complex containing all these ligands or whether they compete with each other for the binding to Yih1, in which case, Yih1 may mediate a cross-talk between different regulatory pathways. In this sense, it will be important to determine the composition of these complexes.

In summary, we propose an additional role for the Gcn2 regulator Yih1. Our results suggest that Yih1 is involved in the cell cycle. Yeast lacking Yih1 shows budding patterns and DNA content distribution indicative of cells accumulating during the G2/M phase of the cell cycle, and this phenotype does not depend on Gcn1 and Gcn2. We also presented several lines of evidences indicating that Yih1 is a *bona fide* Cdc28 binding partner. Interestingly, the interaction between Yih1 and Cdc28 is dynamic and is enhanced when Cdc28 is actively engaged in the cell cycle in association with its regulatory subunits, raising the intriguing possibility that Yih1 could be modulating the activity of Cdc28 in specific stages of the cell cycle, thus accounting for the cell cycle phenotype of *yih1Δ* cells. The mechanisms underlying the role of the Yih1-Cdc28 interaction in this phenotype await further investigation. Furthermore, the orthologue of Yih1, IMPACT, also interacts with CDK1 in mammals and this interaction may have potential implications for the role of IMPACT as a regulator of neuronal differentiation. Finally, these findings provide the basis for future studies on the mechanisms and functions of Yih1 in the cell cycle.

## Supporting Information

S1 File Recombinant His_6_-Cdc28 does not bind to GST-Yih1. Increasing concentrations of His_6_-Cdc28 expressed in *E*. *coli* (1, 2 or 4 μg) were immobilized on Ni-NTA resin. Beads were then incubated with WCEs derived from a wild type strain (MSY-WT2) expressing GST-Yih1 or GST alone under the control of a galactose-inducible promoter. Ni-NTA beads alone were used as negative control. After several washings, proteins bound to His_6_-Cdc28 were subjected to SDS-PAGE and immunoblotted to detect the indicated proteins. Where indicated (+), we incubated recombinant His_6_-Cdc28 or Ni-NTA beads alone with yeast WCEs expressing GST or GST-Yih1. We found that GST-Yih1 from yeast WCEs does not specifically precipitate with His_6_-Cdc28. GST-Yih1 bands shown in immunoblot are a result of nonspecific binding of His_6_-Cdc28 to Ni-NTA beads (compare lanes 4 with lanes 8, 9 and 10). Endogenous Cdc28 precipitates strongly with GST-Yih1 but not with GST-alone (black arrow, lanes 5 to 10). Inputs are shown in lanes 1 and 2. Recombinant His_6_-Cdc28 (4 μg) was loaded as a positive control (lane 11).
(TIF)

S2 File Fluorescence images of yeast cells expressing the VN-tagged Yih1 or the VC-tagged Cdc28 alone. Representative fluorescence images of live cells grown in SD medium to log phase. Strains expressing only one of the tagged protein versions: Yih1-VN, strain VN_3198, or Cdc28-VC, strain BCY21, (panels 1 and 2, respectively) were used as a negative control and showed no detectable fluorescence signal. DAPI staining (blue), merged images and DIC are shown.
(TIF)

S1 Table Yeast strains used in this study. (PDF)

S2 Table Plasmids used in this study. (PDF)
