# Supplementary figures and images for: The Gcn2 Regulator Yih1 Interacts with the Cyclin Dependent Kinase Cdc28 and Promotes Cell Cycle Progression through G2/M in Budding Yeast
Source: PLoS One. 2015 Jul 15;10(7):e0131070. doi: 10.1371/journal.pone.0131070 (PMC4503747; doi:10.1371/journal.pone.0131070)

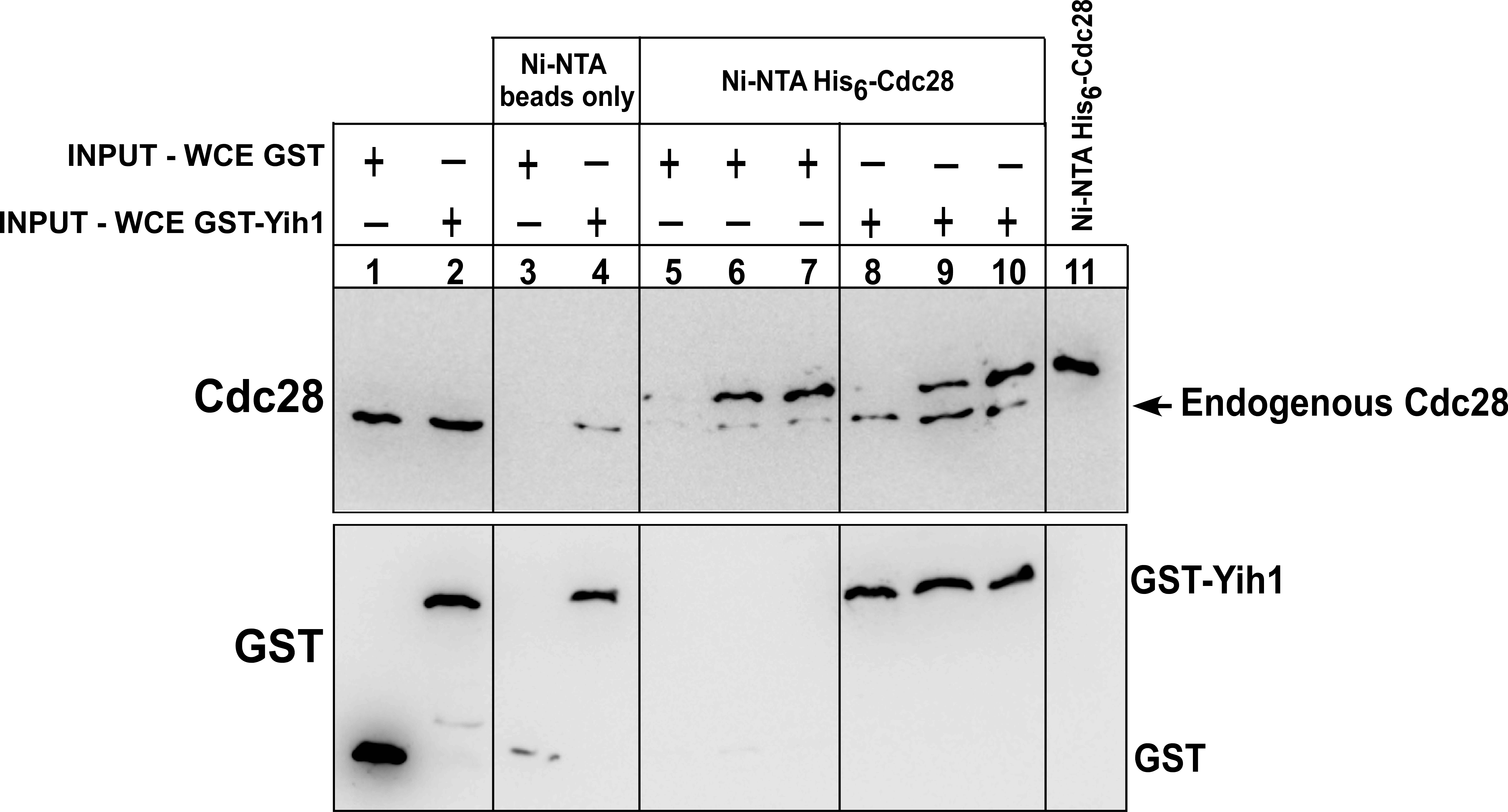

Supplement: S1 File — Increasing concentrations of His6-Cdc28 expressed in E. coli (1, 2 or 4 μg) were immobilized on Ni-NTA resin. Beads were then incubated with WCEs derived from a wild type strain (MSY-WT2) expressing GST-Yih1 or GST alone under the control of a galactose-inducible promoter. Ni-NTA beads alone were used as negative control. After several washings, proteins bound to His6-Cdc28 were subjected to SDS-PAGE and immunoblotted to detect the indicated proteins. Where indicated (+), we incubated recombinant His6-Cdc28 or Ni-NTA beads alone with yeast WCEs expressing GST or GST-Yih1. We found that GST-Yih1 from yeast WCEs does not specifically precipitate with His6-Cdc28. GST-Yih1 bands shown in immunoblot are a result of nonspecific binding of His6-Cdc28 to Ni-NTA beads (compare lanes 4 with lanes 8, 9 and 10). Endogenous Cdc28 precipitates strongly with GST-Yih1 but not with GST-alone (black arrow, lanes 5 to 10). Inputs are shown in lanes 1 and 2. Recombinant His6-Cdc28 (4 μg) was loaded as a positive control (lane 11). (TIF) [file pone.0131070.s001.tif]

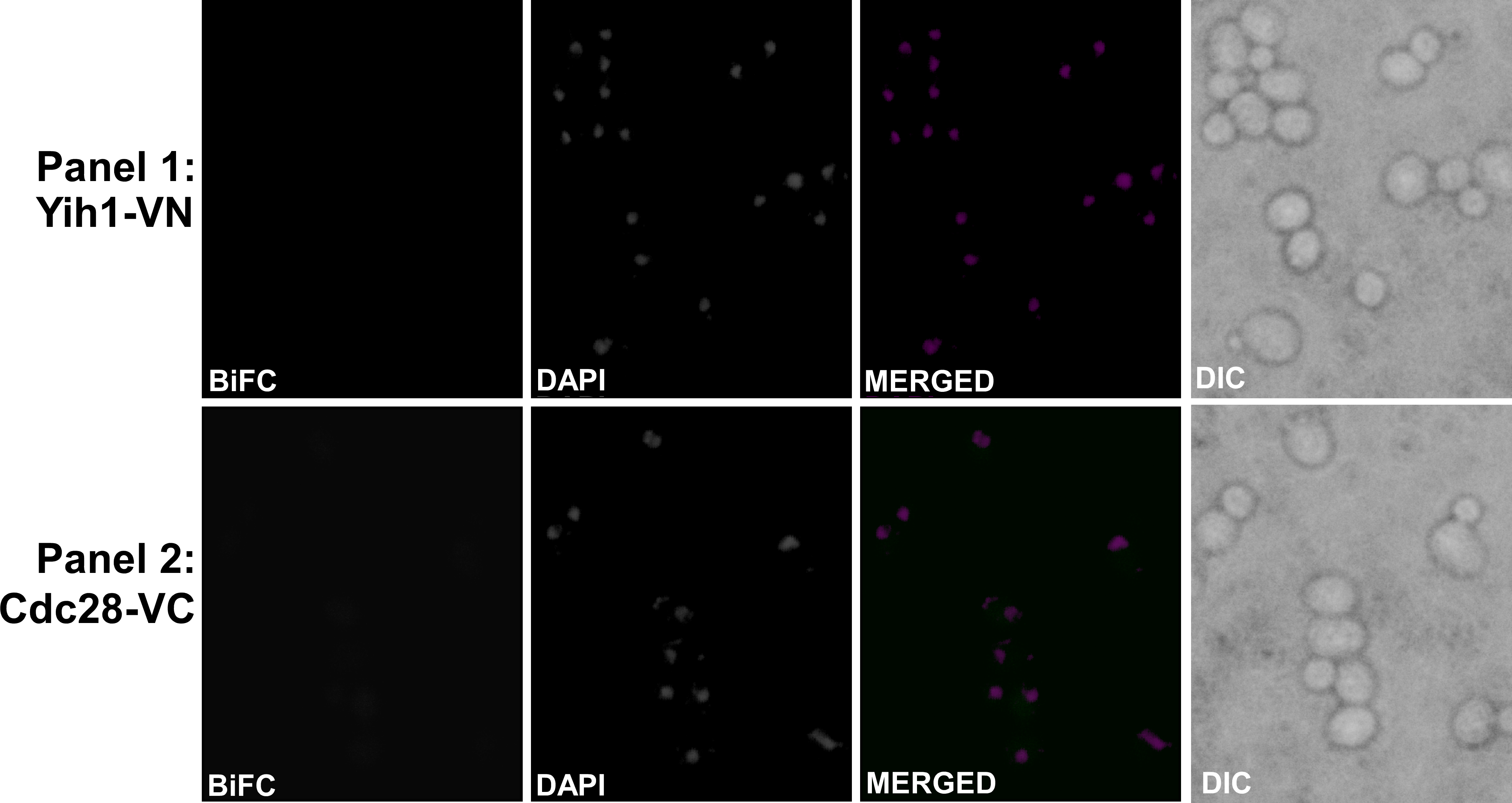

Supplement: S2 File — Representative fluorescence images of live cells grown in SD medium to log phase. Strains expressing only one of the tagged protein versions: Yih1-VN, strain VN_3198, or Cdc28-VC, strain BCY21, (panels 1 and 2, respectively) were used as a negative control and showed no detectable fluorescence signal. DAPI staining (blue), merged images and DIC are shown. (TIF) [file pone.0131070.s002.tif]
