# Supplementary material for: The Gcn2 Regulator Yih1 Interacts with the Cyclin Dependent Kinase Cdc28 and Promotes Cell Cycle Progression through G2/M in Budding Yeast
Source: PLoS One. 2015 Jul 15;10(7):e0131070. doi: 10.1371/journal.pone.0131070 (PMC4503747; doi:10.1371/journal.pone.0131070)
Supplement: S1 Table — (PDF) [file pone.0131070.s003.pdf]

Supplementary Table S1 – Yeast strains used in this study

Strain	Genotype	Source
Genetic background: BY4741		
MSY-WT2	*MATa leu2Δ met15Δ ura3Δ0*	[13]
MSY-Y2	*MATa leu2Δ met15Δ ura3Δ0 yih1Δ*::*Kan*^{R}	[13]
ESY-11b	*MATa leu2Δ met15Δ ura3Δ0 yih1*::*Kan*^{R}*Δ::YIH1-URA3*	[13]
VN_3198	*MATa leu2Δ his3Δ met15Δ ura3Δ0 YIH1-VN::kIURA3*	Korea Bioneer
VN_1325	*MATa leu2Δ his3Δ met15Δ ura3Δ0 CET1-VN::kIURA3*	Korea Bioneer
E57	*MATa ura3-52, lys2-801, ade2-101, trp1Δ1, his3Δ200, leu2Δ1*	[66]
BCY20	As VN_3198, and *CDC28-VC::his3MX6*	This study
BCY21	As E57, and *CDC28-VC::his3MX6*	This study
RRY62a	As VN_3198 and *GCN1-VC*::*his3MX6*	(Ramesh and Sattlegger unpublished)
YBR160W	*MATA leu2Δ his3Δ met15Δ ura3Δ0 CDC28-GFP::his3MX6*	Life Technologies
Genetic background: H1511		
H1511	*MATα ura3-52 trp1-63 leu2-3,112 GAL2+*	[67]
ESY11001b	*As H1511, and yih1Δ*::*Kan*^{R}	[18]
H2556	*As H1511, and gcn1Δ*	[12]
H2557	*As H1511, and gcn2Δ*	[12]
ESY11071aa	*As H1511, and gcn1Δ; yih1*::*Kan*^{R}	[18]
ESY10075aa	*As H1511, and gcn2Δ; yih1*::*Kan*^{R}	This study

66. Sikorski RS, Hieter P. A system of shuttle vectors and yeast host strains designed for efficient manipulation of DNA in Saccharomyces cerevisiae. Genetics. 1989;122(1):19-27. PubMed PMID: WOS:A1989U362600003.
67. Foiani M, Cigan AM, Paddon CJ, Harashima S, Hinnebusch AG. GCD2, a translational repressor of the GCN4 gene, has a general function in the initiation of protein synthesis in Saccharomyces cerevisiae. Molecular and Cellular Biology. 1991;11(6):3203-16. PubMed PMID: 2038326.
