# Supplementary material for: The Gcn2 Regulator Yih1 Interacts with the Cyclin Dependent Kinase Cdc28 and Promotes Cell Cycle Progression through G2/M in Budding Yeast
Source: PLoS One. 2015 Jul 15;10(7):e0131070. doi: 10.1371/journal.pone.0131070 (PMC4503747; doi:10.1371/journal.pone.0131070)
Supplement: S2 Table — (PDF) [file pone.0131070.s004.pdf]

Supplementary Table S2- Plasmids used in this study

Plasmid	Gene	Features	Vector	Source
**Bacterial gene fusion**				
pBE718	GST-YIH1	Amp^R	pGEX-6p-3	This study
RCB14	His_6-Cdc28	Amp^R	pETduet-1	This study
**Yeast gene fusions**				
pES245-6	*GST – YIH1* (2-132)	Amp^R, URA3, 2μ	pES128-9	[3]
pES246-7	*GST – YIH1* (2-171)	Amp^R, URA3, 2μ	pES128-9	[3]
pES247-8	*GST – YIH1* (68-258)	Amp^R, URA3, 2μ	pES128-9	[3]
pES248-9	*GST – YIH1* (68-171)	Amp^R, URA3, 2μ	pES128-9	[3]
pES249-10	*GST – YIH1* (133-258)	Amp^R, URA3, 2μ	pES128-9	[3]
pES187B1	*GST – YIH1* (2-258)	Amp^R, URA3, 2μ	pES128-9	[3]
pES330-5-3	*GST – YIH1* (2-258) E87A; D90A	Amp^R, URA3, 2μ	pES128-9	[3]
pES332-3-1	*GST – YIH1* (2-258) D102A; E106A	Amp^R, URA3, 2μ	pES128-9	[3]
pES234-6-2	*GST – IMPACT*	Amp^R, URA3, 2μ	pES128-9	[14]
**Mammalian gene fusion**				
pBE626	IMPACT-FLAG	Amp^R	pFLAG-CMV5a	(Roffe an Castilho, unpublished)

**Number in parentheses indicate amino acids encoded by Yih1*
